# Insights into the Metabolism and Evolution of the Genus *Acidiphilium*, a Typical Acidophile in Acid Mine Drainage

**DOI:** 10.1128/mSystems.00867-20

**Published:** 2020-11-17

**Authors:** Liangzhi Li, Zhenghua Liu, Min Zhang, Delong Meng, Xueduan Liu, Pei Wang, Xiutong Li, Zhen Jiang, Shuiping Zhong, Chengying Jiang, Huaqun Yin

**Affiliations:** a School of Minerals Processing and Bioengineering, Central South University, Changsha, China; b Key Laboratory of Biometallurgy of Ministry of Education, Central South University, Changsha, China; c State Key Laboratory of Microbial Resources, Institute of Microbiology, Chinese Academy of Sciences, Beijing, China; d College of Zijin Mining, Fuzhou University, Fuzhou, China; e National Key Laboratory of Comprehensive Utilization of Low-Grade Refractory Gold Ores, Shanghang, China; ExxonMobil Research and Engineering

**Keywords:** acid mine drainage, evolution, horizontal gene transfer, comparative genomics, *Acidiphilium*

## Abstract

Here, we report three new *Acidiphilium* genomes, reclassified existing *Acidiphilium* species, and performed the first comparative genomic analysis on *Acidiphilium* in an attempt to address the metabolic potential, ecological functions, and evolutionary history of the genus *Acidiphilium*. In the genomes of *Acidiphilium*, we found an abundant repertoire of horizontally transferred genes (HTGs) contributing to environmental adaption and metabolic expansion, including genes conferring photosynthesis (*puf, puh*), CO_2_ assimilation (*rbc*), capacity for methane metabolism (*mmo, mdh*, *frm*), nitrogen source utilization (*nar, cyn, hmp*), sulfur compound utilization (*sox, psr, sqr*), and multiple metal and osmotic stress resistance capacities (*czc, cop, ect*). Additionally, the predicted donors of horizontal gene transfer were present in a cooccurrence network of *Acidiphilium*. Genome-scale positive selection analysis revealed that 15 genes contained adaptive mutations, most of which were multifunctional and played critical roles in the survival of extreme conditions. We proposed that *Acidiphilium* originated in mild conditions and adapted to extreme environments such as acidic mineral sites after the acquisition of many essential functions.

**IMPORTANCE** Extremophiles, organisms that thrive in extreme environments, are key models for research on biological adaption. They can provide hints for the origin and evolution of life, as well as improve the understanding of biogeochemical cycling of elements. Extremely acidophilic bacteria such as *Acidiphilium* are widespread in acid mine drainage (AMD) systems, but the metabolic potential, ecological functions, and evolutionary history of this genus are still ambiguous. Here, we sequenced the genomes of three new *Acidiphilium* strains and performed comparative genomic analysis on this extremely acidophilic bacterial genus. We found in the genomes of *Acidiphilium* an abundant repertoire of horizontally transferred genes (HTGs) contributing to environmental adaption and metabolic ability expansion, as indicated by phylogenetic reconstruction and gene context comparison. This study has advanced our understanding of microbial evolution and biogeochemical cycling in extreme niches.

## Abstract (precis)

Extremophiles, organisms that thrive in extreme environments, are key models for research on biological adaption. They can provide hints for the origin and evolution of life, as well as improve the understanding of biogeochemical cycling of elements. Extremely acidophilic bacteria such as *Acidiphilium* are widespread in acid mine drainage (AMD) systems, but the metabolic potential, ecological functions, and evolutionary history of this genus are still ambiguous. Here, we sequenced the genomes of three new *Acidiphilium* strains and performed comparative genomic analysis on this extremely acidophilic bacterial genus. We found in the genomes of *Acidiphilium* an abundant repertoire of horizontally transferred genes (HTGs) contributing to environmental adaption and metabolic ability expansion, as indicated by phylogenetic reconstruction and gene context comparison. This study has advanced our understanding of microbial evolution and biogeochemical cycling in extreme niches.

## INTRODUCTION

Prokaryotes occupy almost all environmental niches and have dominated the majority of Earth’s evolutionary history. Extremophiles that thrive in extreme environments represent a key research field in many disciplines, ranging from the adaption to extreme conditions to the cycling of elements in biogeochemistry. Extremophiles also have important implications for the research on the origin of life and the search for life on other planets (1, 2). Acid mine drainage (AMD), characterized by extreme acidity and high concentrations of metals and sulfate, represents an extreme ecological condition and a major global challenge (3). The primary microbial taxa in AMD include *Acidiphilium*, *Acidisphaera*, *Acidithiobacillus*, and *Leptospirillum* (4). The biological factors that contribute to the formation of this hyperacidic environment as well as the adaptive mechanisms of the organisms inhabiting it are hot topics in current research (3, 5). The genus *Acidiphilium* belongs to the family *Acetobacteraceae*, class *Rhodospirillales*, and appears frequently in AMD environments (6, 7). Members of this genus are Gram-negative, photosynthetic, aerobic and facultative anaerobic, metal-respiring, acidophilic heterotrophs (8–10). They grow at pH 1.5 to 7.5, are able to utilize a wide range of organic and inorganic substrates, and synthesize poly-β-hydroxybutyrate (PHB) for carbon storage (11–13). *Acidiphilium* can also resist multiple harmful stressors such as toxic metals (e.g., Cd, Ni, Cr) and osmotic pressure (14–16). There has also been increased interest in the application of *Acidiphilium* spp. for microbial fuel cells (MFCs) (17), as well as in metal mobilization from minerals or waste both in pure culture and in coculture (18–20). Nevertheless, little is known about whether there are divergences in the functional potential and niche partitioning among *Acidiphilium-*affiliated species. In addition, the evolutionary history of many notable properties such as carbon assimilation and metal resistance in *Acidiphilium* is still elusive. *Acidiphilium* is one of only four genera in the family *Acetobacteraceae* found in acidic mineral sites, with the other three genera being *Acidicaldus*, *Acidisphaera*, and *Acidocella* (21–24). Evolutionary drivers such as horizontal gene transfer (HGT) and selection pressure might have played their parts in the adaptive evolution of *Acidiphilium* that survives in harsh acidic mineral conditions. However, their relative contributions are still ambiguous. Horizontal gene transfer refers to the acquisition of genetic elements from distant lineages for genetic and phenotypic innovations, a process contributing significantly to evolution within challenging environments and during global geologic and/or climatic events (25, 26). Positive selection, on the other hand, mediating survival fitness by adaptive mutations, has also been an indispensable driving force in microbial evolution, and recent investigations have shifted from testing selection on individual genes to the entire genomes (27–30).

To assess the differences in metabolic capacity and niche adaption potential among *Acidiphilium* species, and to unravel the evolutionary history of many fundamental genetic properties of *Acidiphilium*, we performed whole-genome sequencing of three novel strains of *Acidiphilium* isolated from two different AMD sites. Comparative genome analysis was carried out, focusing on understanding the roles of evolutionary processes in shaping the genomes of *Acidiphilium*. For this purpose, we conducted a detailed comparison of *Acidiphilium* species. We performed ancestral genomic reconstruction, cooccurrence analysis, and extensive phylogenetic analyses and explored the genomic arrangements of pathways of interest. We also focused on discovering genes under positive selection.

## RESULTS

### Genomic features and reclassification of *Acidiphilium*.

Three *Acidiphilium* genomes (AccI, AccII, and ZJSH63) were sequenced, resulting in a complete genome of strain AccI (a single chromosome and seven plasmids) and high-quality drafts of strains AccII and ZJSH63, according to the MISAG standards (31). The characteristics of these genomes and the other publicly available genomes of *Acidiphilium* spp. used in this study are shown in Table 1. The visualization of strain AccI chromosome (applying colors based on clusters of orthologous group [COG] classes) and comparative analysis of our three genomes were performed (see Fig. S1 at https://doi.org/10.6084/m9.figshare.12892016.v1). The genome size of *Acidiphilium* was about 4 Mbp. Although strains AccI and AccII were isolated from the same site, strain AccI shared more gene families with strain ZJSH63 than AccII. Strain AccII contained the most unique gene families among our three strains. COG annotations showed that AccII contained more unique genes with adaptive functions than ZJSH63 and AccI, especially those related to COG category L (replication, recombination, and repair) and COG N (cell motility). State-of-the-art whole-genome average nucleotide identity (ANI) analysis (32) classified all *Acidiphilium* genomes into four clades (species) based on an ANI cutoff of 95% (see Fig. S2 at https://doi.org/10.6084/m9.figshare.12892016.v1). We found some disagreement between our ANI results and previous nomenclatures of the *Acidiphilium* strains (mainly based on 16S rRNA sequences) in GenBank/JGI-IMG databases (33–35). For example, our ANI results showed that strains of Acidiphilium cryptum and Acidiphilium multivorum, as well as Acidiphilium angustum and Acidiphilium rubrum, should be classified as the same species (ANI > 95%). The major problem with previous species classification, based on 16S rRNA gene sequencing, was the low resolution, as shown by the low bootstrap values of the phylogenetic tree constructed with 16S rRNA sequences (see Fig. S3B at https://doi.org/10.6084/m9.figshare.12892016.v1). This shortage might be overcome by ANI analysis (32). Thus, the genomes of *Acidiphilium* spp. were thereafter referred to as clades I to IV according to their new classification based on ANI (Table 1; see Fig. S2 at https://doi.org/10.6084/m9.figshare.12892016.v1). Strain CAG727 (GCA_000437515.1) was determined not to be a member of *Acidiphilium*, given that it was phylogenetically distant from other *Acidiphilium* strains (see Fig. S2 and S3 at https://doi.org/10.6084/m9.figshare.12892016.v1), and was therefore excluded from further analyses. The clustering of *Acidiphilium* strains based on ANI values was mostly congruent with their geographic locations. For example, strains of clades I, II, and III were isolated from North America, while strains of clade IV were isolated from Europe and East Asia (see Fig. S2 at https://doi.org/10.6084/m9.figshare.12892016.v1). Whole-genome synteny analysis of all available complete sequences of *Acidiphilium* (strains AccI, JF-5, and AIU301, which belong to the same clade) showed that nine conserved locally colinear blocks (LCBs) were present in these strains, but they differed in their order of arrangement and similarity (see Fig. S4A in https://doi.org/10.6084/m9.figshare.12892016.v1). A similarity-based whole-genome comparison of *Acidiphilium* spp. with strain AccI as the reference showed that many genomic regions were not common to all isolates, many of which harbored hypothetical proteins and mobile genetic elements (see Fig. S5 at https://doi.org/10.6084/m9.figshare.12892016.v1).

**TABLE 1:** General features of bacterial genomes used in this study

Organism and strain	GenBank/IMG-ER accession no.	Level	Contig	*N*_50_ (bp)	No. of plasmids	Completeness (%)	Size (Mb)	Coding density (%)	GC (%)	Clade assigned	No. of genes	No. of proteins	Source	Geographic location
*Acidiphilium* sp. strain ZJSH63	2828882166	Draft	301	124,801		95.3	4.39	90.6	66.5	IV	4,330	4,245	Acid mine drainage	Fujian, China
*Acidiphilium* sp. strain AccI	2824045439	Complete	1	4,058,204	7	100	4.18	90.6	66.7	IV	4,304	4,224	Acid mine drainage	Guangdong, China
*Acidiphilium* sp. strain AccII	2824049744	Draft	716	93,659		98.0	4.69	89.4	65.5	IV	4,352	4,347	Acid mine drainage	Guangdong, China
Acidiphilium angustum ATCC 35903	GCA_000701585.1/2561511102	Draft	206	71,968		98.5	4.07	89.6	63.6	II	3,851	3,731	Acid mine	
Acidiphilium cryptum JF-5	GCA_000016725.1/640427101	Complete	1	3,389,227	8	100	3.96	90.8	67.1	IV	3,747	3,574	Acid mine	
Acidiphilium multivorum AIU301	GCA_000202835.1	Complete	1	3,749,411	8	100	4.21	89.8	67.0	IV	3,991	3,803	Acid mine water	Iwate, Matsuo, Japan
Acidiphilium rubrum ATCC 35905	GCA_900156265.1/2681812815	Draft	78	136,338		98.6	3.98	89.9	63.7	II	3,752	3,692	Acidic coal mine drainage	Pennsylvania, USA
*Acidiphilium* sp. strain 20-67-58	GCA_002255515.1	Draft	119	80,126		98.6	3.41	89.4	66.6	III	3,199	3,073	Mine wastewater	Ontario, Canada
*Acidiphilium* sp. strain 21-60-14	GCA_002255745.1	Draft	132	63,048		98.6	3.06	90.8	60.2	I	2,940	2,828	Mine wastewater	Ontario, Canada
*Acidiphilium* sp. strain 21-62-4	GCA_002255545.1	Draft	433	1,584		16.2	0.69	85.0	61.7	II	910	832	Mine wastewater	Ontario, Canada
*Acidiphilium* sp. strain 21-66-27	GCA_002255645.1	Draft	542	1,752		11.5	1.02	82.6	65.7	III	1,316	1,189	Mine wastewater	Ontario, Canada
*Acidiphilium* sp. strain 21-68-69	GCA_002279225.1	Draft	884	2,201		44.6	1.81	82.3	67.9	III	2,291	1,999	Mine wastewater	Ontario, Canada
*Acidiphilium* sp. strain 34-60-192	GCA_002282645.1	Draft	89	65,514		83.8	3.11	90.6	60.1	I	3,029	2,695	Mine wastewater	Ontario, Canada
*Acidiphilium* sp. strain 34-64-41	GCA_002282635.1	Draft	180	38,599		93.9	3.86	88.6	63.7	II	3,623	3,413	Mine wastewater	Ontario, Canada
*Acidiphilium* sp. strain 37-60-79	GCA_002279355.1	Draft	98	72,002		96.6	3.07	90.9	60.0	I	2,916	2,791	Mine wastewater	Ontario, Canada
*Acidiphilium* sp. strain 37-64-53	GCA_002279345.1	Draft	211	43,062		97.3	4.03	88.3	63.5	II	3,799	3,620	Mine wastewater	Ontario, Canada
*Acidiphilium* sp. strain 37-67-22	GCA_002279335.1	Draft	937	3,121		62.2	2.48	89.9	67.0	III	2,942	2,632	Mine wastewater	Ontario, Canada
*Acidiphilium* sp. strain bin8_M5	2734482270	Draft	74	76,901		97.3	3.08	92.7	68.8	IV	3,042	2,992	Acid mine drainage	Guangdong, China
*Acidiphilium* sp. strain CAG727	GCA_000437515.1	Draft	116	9,562		82.5	1.73	—^*a*^	45.4	—	1,509	1,479	Gut microbiota	
*Acidiphilium* sp. strain JA12-A1	GCA_000724705.2/2571042905	Draft	296	44,500		98.6	4.18	88.4	66.9	IV	4,059	3,719	Acid mine drainage	Lusatia, Germany, Europe
*Acidiphilium* sp. strain PM	GCA_000219295.2	Draft	627	12,446		91.2	3.93	86.4	66.4	IV	3,908	3,859	Acidic, metal-rich water	Rio Tinto, Spain, Europe

a —, not available.

### Core genome and pangenome of *Acidiphilium*.

**FIG 1:**
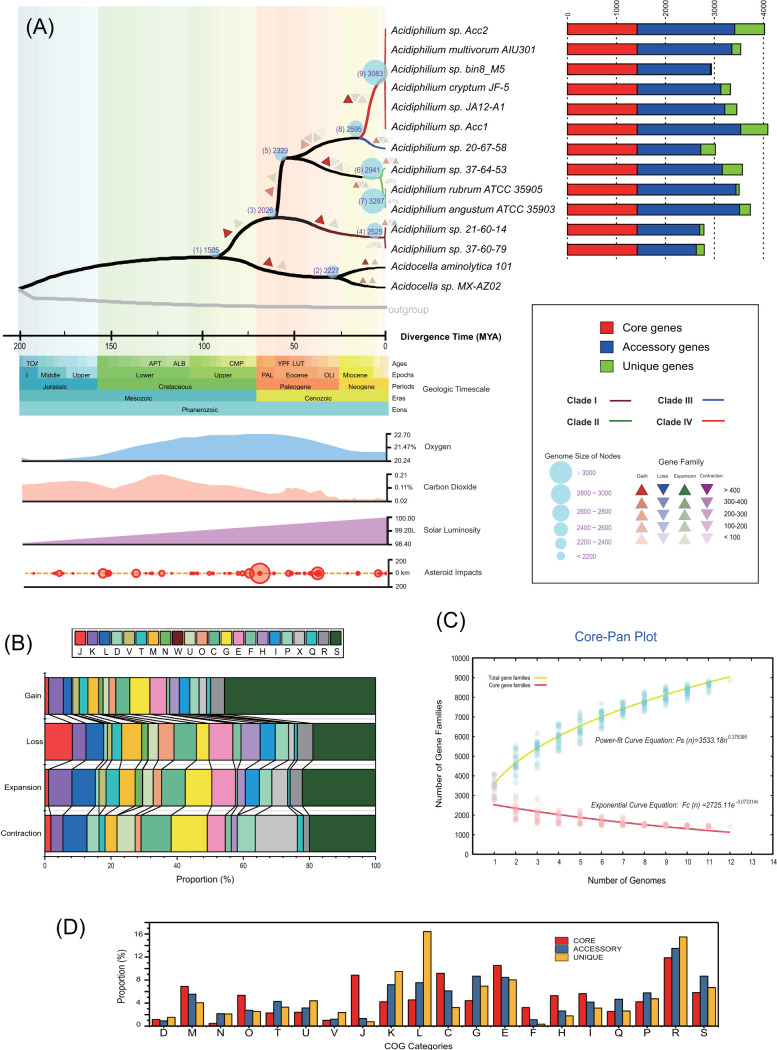
(A) The evolutionary timeline of *Acidiphilium* was estimated (left) using RelTime on top of the rooted NJ tree based on the concatenated alignment of 133 core genes. Ancestral genome content reconstruction of *Acidiphilium* was performed with Count software, and the color depth represents the numbers of reconstructed gain, loss, expansion, and contraction events of each lineage. Data of asteroid impacts, solar luminosity, and fluctuations in atmospheric oxygen and carbon dioxide concentrations are displayed synchronously with divergence times in the form of time panels (left). A stack bar diagram (right) shows the number of genes shared by all strains (i.e., the core genome), the number of genes shared by partial strains (i.e., the accessory genome), and the number of strain-specific genes (i.e., the unique gene) in the tested strains. (B) Stack bar chart showing functional proportions of *Acidiphilium* gene families undergoing gain, loss, expansion, and contraction events as based on COG classes. (C) Mathematical modeling of the pangenome and core genome of *Acidiphilium.* (D) Bar chart showing functional proportions (based on COG categories) of different parts of the *Acidiphilium* pangenome (i.e., core, accessory, unique). Detailed descriptions of the COG categories are provided in Text S1 in the supplemental material.

**FIG 2:**
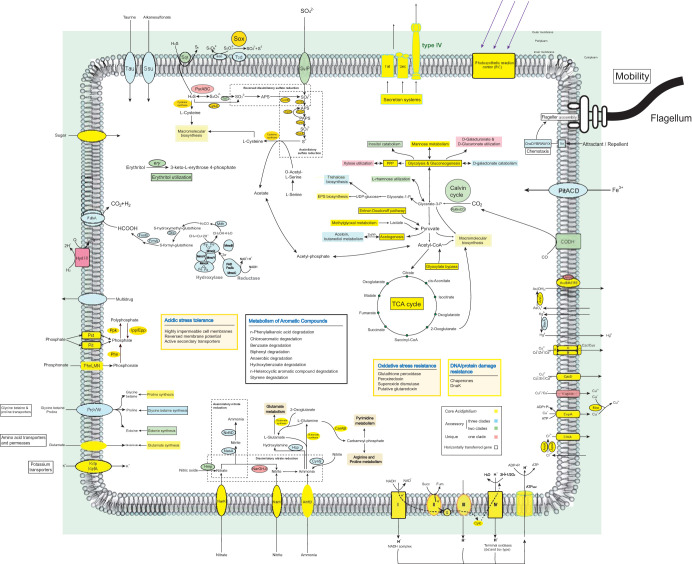
Overview of metabolic potentials in *Acidiphilium* as predicted from genome annotation; core/specific metabolic features are shown with different colors, and pathways containing predicted horizontally transferred genes are marked with black rectangles.

Twelve genomes of the genus *Acidiphilium*, with estimated completeness over 97.0%, were carefully chosen for further pangenome analysis and genomic content reconstruction. The phylogenetic trees based on the concatenated alignment of 133 core genes inferred with neighbor-joining (NJ) methods were congruent with that based on whole-genome sequences (Fig. 1A; see Fig. S3A at https://doi.org/10.6084/m9.figshare.12892016.v1). The pangenome of the 12 *Acidiphilium* strains possessed 8,845 gene families, while the core genome possessed 1,422 gene families accounting for only 16.1% of the pangenome (Fig. 1C). Core and pangenome analyses of the 12 *Acidiphilium* genomes revealed an ‘‘open’’ pangenome fitted into a power-law regression function [*P_s_* (*n*) = 3,533.18*n*^0.375395^], while the core genome was fitted into an exponential regression [*F_c_* (*n*) *=* 2,725.11*e* ^−0.073314^*^n^*] (Fig. 1A). The open pangenome suggested that species have undergone considerable gene exchanging to extend their functional profiles (36). Functional COG annotation revealed that the core genome had a higher proportion of genes classified in COG categories J (translation, ribosomal structure, and biogenesis), C (energy production and conversion), O (posttranslational modification, protein turnover, chaperones), F (nucleotide transport and metabolism), and H (coenzyme transport and metabolism), all associated with basic biological functions. The accessory genome and strain-specific genes were biased toward COG categories G (carbohydrate transport and metabolism), L (replication, recombination, and repair), P (inorganic ion transport and metabolism), and N (cell motility) (Fig. 1D), which were probably related to the adaption of *Acidiphilium* to oligotrophic, metal-laden, and acidic environments that often cause DNA damage. Gene ontology (GO) enrichment analyses produced similar results (see Table S1 in the supplemental material). Detailed metabolic reconstruction of *Acidiphilium* was also performed; the core/specific metabolic features are shown in Fig. 2 using different colors, and pathways containing predicted horizontally transferred genes are marked with black rectangles.

10.1128/mSystems.00867-20.1 TABLE S1 GO enrichment analyses (*P* value < 0.05) of *Acidiphilium* gene families. Download Table S1, XLSX file, 0.02 MB. Copyright © 2020 Li et al. 2020 Li et al. This content is distributed under the terms of the Creative Commons Attribution 4.0 International license.

10.1128/mSystems.00867-20.6 TEXT S1 Detailed description for abbreviations of COG categories. Download Text S1, DOCX file, 0.01 MB. Copyright © 2020 Li et al. 2020 Li et al. This content is distributed under the terms of the Creative Commons Attribution 4.0 International license.

### MGEs and CRISPR-Cas systems.

Mobile genetic elements (MGEs), such as insertion sequences, transposases, genomic islands (GIs), plasmids, and phages, are known signals of HGT events, and the number of MGEs correlates positively with the frequency of HGT (37). MGEs in the genomes of *Acidiphilium* were identified in this study (Table S2). The average number of transposon sequences per genome was 307, with *A. multivorum* AIU301 harboring the greatest number (841). Members of the ISAli7, ISGalb1, ISMex27, ISAan1, and ISAcr4 families were most common. The average number of sequences located in GIs per genome was 527, with *Acidiphilium* sp. strain ZJSH63 containing the most (1,055). The average number of prophages and/or prophage remnants per genome was 23, with *Acidiphilium* sp. strain AccII harboring the most (58, with a total size of 69.9 kb). The number of plasmids in *Acidiphilium* could reach eight (*A. cryptum* JF-5 and *A. multivorum* AIU301). The functional gene profiles of plasmids from these three completely sequenced strains were also compared (see Fig. S4B to D at https://doi.org/10.6084/m9.figshare.12892016.v1). Approximately 66 gene families were shared among plasmids from these three strains, and certain degrees of collinearity were observed. COG L (replication, recombination, and repair) functions were enriched in the plasmid genomes. Type I-C/E/V and II-C CRISPR-Cas systems were also found in *Acidiphilium* spp., with *Acidiphilium* sp. strain PM containing the most CRISPR-Cas-related genes or spacers (38). The abundant MGEs present in genomes of *Acidiphilium* indicated that HGT might have contributed significantly to the genomic evolution of *Acidiphilium* species during niche adaption, while the CRISPR-Cas system would also help protect the genomes of *Acidiphilium* by eliminating harmful genomic intrusion events, balancing genomic stability and functional investments (39). A recent study also revealed that spacer sequences of the CRISPR-Cas system could not only specify the targets of Cas nucleases but also facilitate HGT (40).

10.1128/mSystems.00867-20.2 TABLE S2 Statistics of mobile genetic elements (MGEs) present in the genomes of *Acidiphilium* spp. Download Table S2, XLSX file, 0.5 MB. Copyright © 2020 Li et al. 2020 Li et al. This content is distributed under the terms of the Creative Commons Attribution 4.0 International license.

### Evolutionary analyses of *Acidiphilium*.

The evolutionary timeline of *Acidiphilium* was also estimated on the rooted core gene tree (Fig. 1A). Overall, gene families undergoing gain events outnumbered those undergoing loss events by approximately three times (6,319 versus 2,231), and gene families undergoing expansion events outnumbered those undergoing contraction events by approximately 20 times (1,173 versus 55) in the genomes of *Acidiphilium*. Our analyses suggested that there has been an ongoing increase in genomic content throughout the evolutionary history of this genus, from an estimated 2,026 gene families in the common ancestor to over 3,000 gene families. Predicted gain events of over 400 gene families occurred at nodes 3, 4, 6, and 9, accounting for approximately 14% to 25% of gene families at the corresponding nodes. Of all gene families undergoing gain events, about half encoded hypothetical proteins. A considerable proportion of gain events were related to COG category G (carbohydrate transport and metabolism, 6.1%) and COG category E (amino acid transport and metabolism, 5.0%), and a notable proportion of gene families undergoing expansion events were also related to COG categories G (carbohydrate transport and metabolism, 8.0%), E (amino acid transport and metabolism, 7.2%), C (energy production and conversion, 7.2%), K (transcription, 7.0%), and L (replication, recombination, and repair, 7.0%) (Fig. 1B). It seems that the COG categories involved are carbohydrate metabolism and transport as well as amino acid metabolism and transport, which reflect the adaptive strategies of *Acidiphilium*, including the expansion of metabolic abilities to utilize a variety of potential nutrients, while the acquisition of efficient repair mechanisms is in response to damage of biological molecules possibly caused by harsh environments such as AMD sites. This was in line with previous work which showed that larger genomes preferentially accumulated genes associated with metabolism, regulation, and energy conversion (41). We also found that 8.3% of gene families undergoing loss events belonged to COG category J (translation, ribosomal structure, and biogenesis) and that 12.7% of gene families undergoing contraction events belonged to COG category X (mobilome: prophages, transposons) (Fig. 1B), which were probably related to a holistic adjustment toward a more efficient operational and survival mode of these heterotrophs. The most recent common ancestor (MRCA) of *Acidiphilium* spp. was estimated to have emerged around 60.3 million years ago (Mya) (Fig. 1A, left), not long after a recorded strong asteroid impact, which we postulated to be one of the possible courses of significant changes to the Earth’s atmosphere, since it coincided with a decrease in atmospheric O_2_ and increase in atmospheric CO_2_.

**FIG 3:**
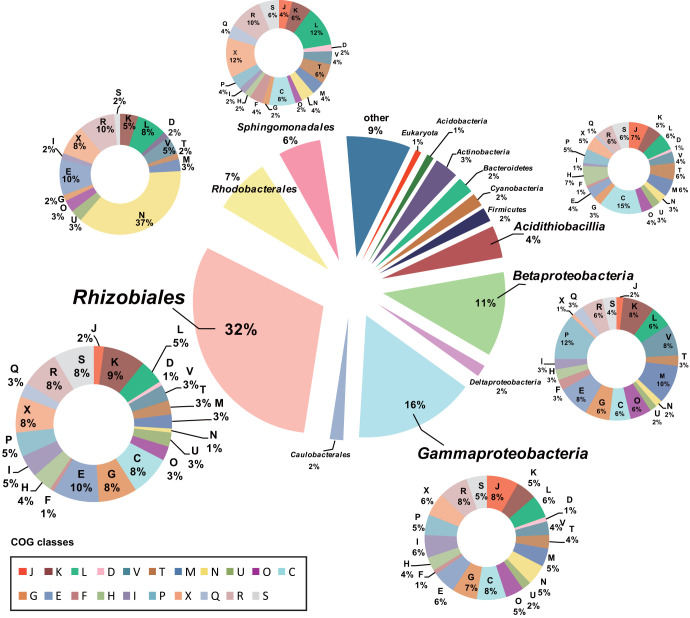
Pie charts showing donors that putatively transferred genes to *Acidiphilium* spp., their relative contributions to total HGT events, and the functional proportions of the HGT functions derived from respective donors based on COG classes. Detailed descriptions of the COG categories are provided in Text S1 in the supplemental material.

**FIG 4:**
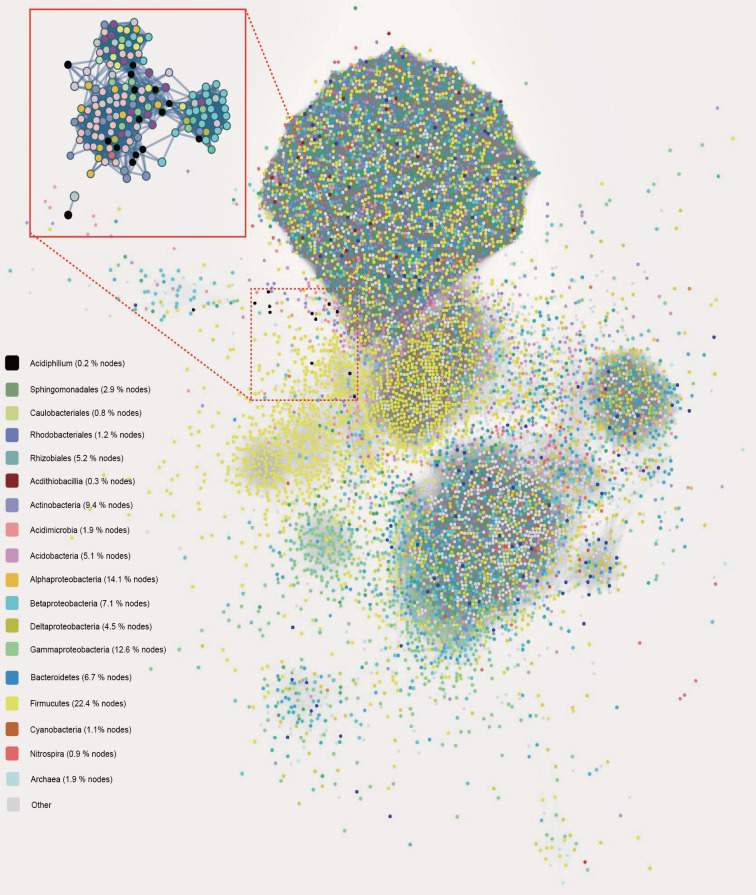
Cooccurrence network based on correlation analysis of 16S rRNA amplicon sequencing data sets of AMD samples (*n* = 205). Each node denotes a microbial OTU at a 97% cutoff. The first neighbors of *Acidiphilium* nodes (highlighted by a red rectangle) were selected using the tool “first neighbors of selected nodes” in Cytoscape.

We extracted HGT events predicted with the IMG Annotation Pipeline (Table S3). Results showed that a notable set of genes were identified as being acquired via HGT, accounting for up to 18.9% of genes among tested *Acidiphilium* genomes, indicating the chimeric nature of these genomes. Cross-order HGT events from *Rhizobiales* were the most frequent, accounting for ∼32% of total HGT events, followed by cross-class HGT events from *Gammaproteobacteria* (∼16%) and *Betaproteobacteria* (∼11%) and cross-order HGT events from *Rhodobacterales* (∼7%) and *Sphingomonadales* (∼6%). A considerable proportion (∼4%) of genes were derived via cross-class HGT from the typical AMD autotroph *Acidithiobacillia* (Fig. 3). The above-mentioned HGT donors were almost all consistently present together with *Acidiphilium* in the cooccurrence network based on 16S rRNA gene amplicon data sets generated from AMD samples (Fig. 4). In the cooccurrence network, *Acidiphilium* accounted for 0.2% of the nodes, while the HGT donors occupied 0.3 to 22.4% of the nodes. Furthermore, most of these HGT donors were present in the first-neighbor network of *Acidiphilium.* Function annotations of putative horizontally transferred genes (HTGs) based on COG classes showed that these genes were biased toward COG categories E (amino acid transport and metabolism), K (transcription), C (energy production and conversion), and G (carbohydrate transport and metabolism), which are associated with metabolic and energy production processes, COG categories L (replication, recombination, and repair) and V (defense mechanisms), all associated with defense and repair mechanism, COG category X (mobilome: prophages, transposons), involved in the mobilization of genome fragments, and COG category M (cell wall/membrane/envelope biogenesis), related to enhanced cellular barriers against external disturbance. COG category N (cell motility) was found to account for up to 37% of *Rhodobacterales*-derived HGT (Fig. 3), which might facilitate *Acidiphilium* to swim away from harmful environments and/or toward nutrients. We further performed detailed analyses of gene synteny and phylogeny for examination and quantification of the cumulative impact of HGT on *Acidiphilium* evolution.

10.1128/mSystems.00867-20.3 TABLE S3 Extracted information of putative horizontally transferred genes from the results of IMG annotation. Download Table S3, XLSX file, 0.04 MB. Copyright © 2020 Li et al. 2020 Li et al. This content is distributed under the terms of the Creative Commons Attribution 4.0 International license.

### Environmental stress adaption.

*Acidiphilium* was predicted to originate at a time when the O_2_ concentration present in the atmosphere reached its peak (22.7%) (Fig. 1A, left). As *Acidiphilium* evolved, the atmospheric O_2_ concentration decreased to about 20.6% in a consistent manner. In contrast, solar luminosity gradually increased to current levels (100 L), and the atmospheric CO_2_ concentrations first increased by approximately 0.1%, followed by a decrease of about 0.05% (Fig. 1A, left). The *bd*-type oxidase encoded by *cydAB* for oxygen-reducing energy production was present in all clades of *Acidiphilium* but was not uniform with gene synteny and discrepancy of phylogeny compared with the species tree (see Fig. S3, S6, and S7 at https://doi.org/10.6084/m9.figshare.12892016.v1). This suggested that independent HGT events contributed to the acquisitions of *cydAB* after the speciation of clades I and II but before the divergence of clades III and IV. The acquisitions of *cydAB* genes were probably in adaption to decreasing atmospheric O_2_ concentrations, considering the high affinity of *bd*-type oxidase even at low O_2_ concentrations (42). Additionally, cytochrome *bo_3_-*type ubiquinol oxidase (CyoABCD), which facilitates growth at low pH and low O_2_ concentrations (43), was found in *Acidiphilium* clades I and IV, which were probably acquired from species sharing a habitat with *Acidiphilium*, such as *Acidithiobacillus*, *Acidihalobacter*, and *Acidiferrobacter* species (see Fig. S8 at https://doi.org/10.6084/m9.figshare.12892016.v1). The *nuo* gene cluster in *Acidiphilium* that encodes NADH:ubiquinone oxidoreductase (complex I), and functions preferentially under aerobic conditions (44–46), was also HGT derived (see Fig. S9 at https://doi.org/10.6084/m9.figshare.12892016.v1). These acquisitions might explain the facultative anaerobic ability of *Acidiphilium*. The HGT-derived Calvin cycle-related gene cluster *prk-rbcLS-cbbX* was present in clades II and IV of *Acidiphilium* (see Fig. S10 to S14 at https://doi.org/10.6084/m9.figshare.12892016.v1), which might help *Acidiphilium* overcome oligotrophic AMD conditions through CO_2_ assimilation. Carbon monoxide dehydrogenase HTGs (CoxLMS) were detected in clades III and IV (see Fig. S15 and S16 at https://doi.org/10.6084/m9.figshare.12892016.v1). This suggested that *Acidiphilium* might utilize CO that was present in mine areas (47) as an energy supplement and source of CO_2_, since the atmospheric CO_2_ concentration dropped to a lower level upon the diversification of *Acidiphilium* (Fig. 1A, left). Photosystem II-type photosynthetic reaction centers (PufBALMC and PuhA) were found in all clades of *Acidiphilium*, probably transferred to *Acidiphilium* after the speciation of clade III (see Fig. S17 to S19 at https://doi.org/10.6084/m9.figshare.12892016.v1), coinciding with the acquisition of gene clusters *prk-rbcLS-cbbX* and *coxLMS.* Ni/Fe hydrogenase HTGs were detected only in clade IV (see Fig. S20 at https://doi.org/10.6084/m9.figshare.12892016.v1). AMD sites that *Acidiphilium* inhabits are hyperosmotic and rich in various metals due to the corrosion of minerals by sulfuric acid and chemoautotrophic microbes (48). We discovered a set of HGT-derived heavy metal resistance genes as well as osmotic pressure resistance genes in the genomes of *Acidiphilium* (see Fig. S21 to S46 at https://doi.org/10.6084/m9.figshare.12892016.v1). For example, *apcA* (49), *arsH* (50), which encodes an NADPH-dependent metal-reducing cytochrome/protein, *merA*, encoding mercuric reductase, *chrA*, encoding a chromate transporter, and *arsRCB*, which confers arsenic resistance, were present in all clades of *Acidiphilium*. It was notable that two of our strains, AccI and AccII, harbored more copies of ferric ion-reducing *apcA* genes (49) than strain ZJSH63, probably due to the higher ferric ion concentration in the sites that AccI and AccII inhabited. HGT-derived toxic divalent cation (e.g., cadmium, zinc, cobalt, and copper) resistance genes *czcABC*, *czcD*, *copA*, and *mco* and regulatory genes *czrRS* were also present in all clades of *Acidiphilium*, while HGT-derived Cu homeostasis genes *copCD* (51) were detected only in some strains of clade IV. Chelation of metals with polyphosphate (polyP) is also an effective metal resistance mechanism of acidophiles (52). HGT-derived alkaline phosphatases (Alp), which release phosphate groups from various compounds, and phosphate import systems encoded by *pstSACB* and *pitA* were present in all clades of *Acidiphilium*. Compatible solute uptake and biosynthesis as well as potassium uptake are known as common strategies to counteract osmotic stress (53, 54). The HGT-derived biosynthetic pathway of the compatible solute hydroxyectoine conferred by the gene cluster *ectRABCD-ask* (55) was detected in the genomes of clades III and IV. HGT-derived *kdpABCDE*, which confer resistance to osmotic stress by uptake of K^+^, were present in all clades of *Acidiphilium*, likely acquired via individual HGT. HGT-derived gene cluster *proXWV-betBA-soxBDAG*, involved in the uptake and biosynthesis of glycine betaine, is present in all clades of *Acidiphilium* except for clade I. This gene cluster was likely gained before diversification of clades III and IV but after speciation of clade II. A standalone HTG, *betC*, also involved in glycine betaine synthesis, was present only in clades III and IV. Motility and chemotaxis conferred by flagellar and sensing proteins might help microbes swim away from harmful environments and toward favorable chemicals or other nutrients. We found operons involved in flagellar biosynthesis such as *flg*, *flh*, and *fli* located in identified genomic islands (GIs) (Table S2C), and the HGT-derived chemotaxis operon *cheDYBRWAYX* was also found in all clades of *Acidiphilium*.

### Metabolic potential expansion through HGT.

Many parts of the sulfur, nitrogen, and carbon metabolic pathways in *Acidiphilium* were also acquired through HGT. The Sox multienzyme complex (encoded by *soxCDXYZAB* and the regulatory *soxH* and *soxR* genes), which oxidizes thiosulfate to sulfate (56), was present in all clades of *Acidiphilium* and was likely derived via independent HGT events in clade I, clade II, and the MRCA of clades III and IV (see Fig. S47 and S48 at https://doi.org/10.6084/m9.figshare.12892016.v1). HTG *sqr*, encoding sulfide:quinone oxidoreductase, was found in clades III and IV (see Fig. S49 at https://doi.org/10.6084/m9.figshare.12892016.v1). HGT-derived polysulfide reductase (PsrABC) was found in clade IV, while thiosulfate sulfurtransferase (Tst) was detected only in clades II and IV (see Fig. S50 to S52 at https://doi.org/10.6084/m9.figshare.12892016.v1). Homologues of Sdo1 in Acidithiobacillus caldus MTH-04 (A5904_0421), a new sulfur dioxygenase associated with tetrathionate oxidation (57), were detected in all clades of *Acidiphilium* except clade II, likely acquired after the diversification of clades III and IV (see Fig. S53 and S54 at https://doi.org/10.6084/m9.figshare.12892016.v1). Thiosulfate dehydrogenase/tetrathionate reductase (encoded by *tsdA*), which mediates the flow of electrons into respiratory or photosynthetic electron chains (58), was also detected in *Acidiphilium* outside clade I (see Fig. S55 at https://doi.org/10.6084/m9.figshare.12892016.v1), which was likely acquired after the emergence of clade III (see Fig. S56 at https://doi.org/10.6084/m9.figshare.12892016.v1). Reversible assimilatory sulfate reduction (conferred by *cysND* and *cysJHI*), found in all clades of *Acidiphilium*, was inferred to be acquired before diversification of clades III and IV but after the speciation of clade II (see Fig. S57 to S59 at https://doi.org/10.6084/m9.figshare.12892016.v1). Nitrogen metabolism enzymes for nitrate/nitrite transporter (NarK), dissimilatory nitrate reductase (NarGHJI), nitric oxide dioxygenases (Hmp), cyanate lyase (CynS), hydroxylamine reductase (Hcp), assimilatory nitrate reductase (NasA), and nitrite reductase (NirBD) were all derived via HGT. However, they were only sparsely present in *Acidiphilium* (mostly clade IV) (see Fig. S60 to S64 at https://doi.org/10.6084/m9.figshare.12892016.v1).

Abundant HTGs involved in carbon metabolism were also detected, including those coding for a complete methane catabolic pathway and many key genes involved in hydrocarbon utilization. An HGT-derived methane catabolism pathway (conferred by genes *mmoXCYB*, *fdhA*, *mdh*, *gfa*, *frmA*, and *frmB*) was found in almost all clades of *Acidiphilium* (see Fig. S65 to S70 at https://doi.org/10.6084/m9.figshare.12892016.v1). Soluble methane monooxygenase (sMMO) converts methane to methanol, which could be further converted to formaldehyde by methanol dehydrogenase (Mdh). Formaldehyde is eventually converted to CO_2_ via enzymes encoded by *gfa*, *frmA*, and *frmB* (59, 60). Multiple genes related to glycolysis and gluconeogenesis (*fba*, *fbp*, GNL), the pentose phosphate pathway (*tkt*, *tal*, *pgd*, *rpe*, G6PD, *prsA*, and *xfp*), the Entner-Doudoroff pathway (*gdh*, *ddgk*, and *pglD*), methylglyoxal metabolism (*megR*), tricarboxylic acid cycle or glyoxylate bypass (*fumC*, *mdh*, *mdh*, *mls*, *icl*), acetogenesis (*spxB*, *pta*, PDHA1, *actP*), and the previously mentioned *prk-rbcLS-cbbX* involved in the Calvin cycle were proposed to have been acquired via HGT (see Fig. S71 to S92 at https://doi.org/10.6084/m9.figshare.12892016.v1). In addition, the *rha* operon, involved in metabolism of l-rhamnose, was present in the genomes of clades II and IV, likely acquired by HGT (see Fig. S93 at https://doi.org/10.6084/m9.figshare.12892016.v1). For sugar alcohol metabolism, genes involved in the metabolism of erythritol and inositol were found only in clades II and IV. The HGT-derived cluster *iolBDC-inoKEF-iolIG_1_HEG_2_G_3_*, conferring inositol catabolic ability, was detected in clade IV, while a similar but differently arranged gene cluster, *iolDEG_1_G_2_-inoEKF-iolBCG_3_*, was detected in clade II (see Fig. S94 at https://doi.org/10.6084/m9.figshare.12892016.v1). In addition, the *ery* operon, involved in erythritol utilization, was presented in clades II and IV of *Acidiphilium*, likely acquired via cross-order HGT (see Fig. S95 at https://doi.org/10.6084/m9.figshare.12892016.v1).

Multiple genes involved in the biodegradation and metabolism of aromatic compounds showed patchy distribution throughout the genus *Acidiphilium*. Many were gained via HGT (see Fig. S96 to S103 at https://doi.org/10.6084/m9.figshare.12892016.v1), including genes *fadA*, *fadB*, *fadD* (*n*-phenylalkanoic acid degradation), gene cluster *catFIJ* (chloroaromatic degradation), gene cluster *benABCD-catBCA* (benzoate degradation), and genes *pcaGH*, *pcaB*, *pcaC*, *pcaD*, *pcaT*, *pcaQ*, and *pcaIJ* (beta-ketoadipate pathway), together with *pobA* and *pcaK* (hydroxybenzoate degradation), *iqoAB* (*n*-heterocyclic aromatic compound degradation), and *fahA* and *faaH* (styrene degradation).

### Positive selection analyses.

Genome-scale positive selection analyses were exhaustively performed on all 21 genomes of *Acidiphilium* in this study in order to expand the input data set. The complete genome of *Acidiphilium* sp. AccI was used as the anchor genome. Results showed that 15 genes were identified as being under positive selection, including Ap_37 (exoribonuclease), Ap_279 (acyl coenzyme A [acyl-CoA] dehydrogenase), Ap_346 (H^+^/Cl^−^ exchange transporter), Ap_374 (membrane component of nitrite reductase), Ap_696 (hypothetical protein), Ap_835 (*N*-acetyltransferase), Ap_1029 (hypothetical protein), Ap_1038 (Holliday junction resolvase), Ap_1473 (3-hydroxy acid dehydrogenase), Ap_1766 (hypothetical protein), Ap_1855 (asparagine synthetase), Ap_2034 (transposase), Ap_2355 (pyridoxine/pyridoxamine 5′-phosphate oxidase), Ap_2619 (cysteine synthase), and Ap_2636 (thioredoxin) (Table S4).

10.1128/mSystems.00867-20.4 TABLE S4 Genes in *Acidiphilium* identified as being under positive selection using the PosiGene pipeline. Download Table S4, DOCX file, 0.01 MB. Copyright © 2020 Li et al. 2020 Li et al. This content is distributed under the terms of the Creative Commons Attribution 4.0 International license.

## DISCUSSION

In this study, we present a detailed analysis of the metabolic capabilities and evolutionary history of the genus *Acidiphilium*. Abundant HGT events were found to contribute substantially to the genomic contents of *Acidiphilium*, providing this genus with unprecedented elasticity to counteract harsh conditions such as those found in AMD. HGT may also have had a great impact on the diversity of *Acidiphilium* gene repertoires. Genome size dynamics (“Why are some genomes really big and others quite small?”) and the occurrence of horizontal gene transfer (“Why does lateral transfer occur in so many species and how?”) were listed as two world-class scientific questions that awaited answers by the editorial of the journal *Science* entitled “So much more to know” (61), and we believe that the present study might provide some clues for these questions. Our results showed that the genome size of *Acidiphilium* was relatively large (∼4 Mbp), with overwhelming gene family gains predicted across its evolution. This is in sharp comparison with its endosymbiotic *Acetobacteraceae* relative (genome size of ∼2 Mbp) that underwent genome reduction (62, 63). Microbes tend to evolve relatively large genomes with higher nutrient uptake and metabolic potential as a means to compensate for fluctuating and inhospitable environments (64–66). This theory could be applied to *Acidiphilium* spp. that inhabit hyperacidic, metal-laden, nutrient-depleted AMD environments, which are quite different from the stable environments (with plentiful easy-to-metabolize resources) in which their endosymbiotic *Acetobacteraceae* relatives dwell (67). The gene repertoire of microbes might evolve rapidly, with HGT being a major source of gene acquisitions in microbial genomes, and a number of genomic analyses have shown that microorganisms adopted new a “lifestyle” via HGT in the colonization of new niches (68). In addition, HGT occurs mainly in the form of horizontal operon transfer (HOT) (69), since many functional modules require a contiguous gene cluster, as exemplified by the acquisition of the photosynthetic operon in *Rhodobacteraceae* (70). Consistent with this, our results showed that HGT of functional genes (cluster) might have conferred to *Acidiphilium* better environmental adaptations as well as the expansion of a wide range of metabolic abilities. For example, the coincided acquisitions of the photosystem II-type photosynthetic reaction center (RC), Calvin cycle enzymes, and carbon monoxide dehydrogenases might have conferred adaptive benefits to *Acidiphilium* by taking advantage of the high CO levels (∼50 ppm) in mining areas as an energy source (47, 71) and the increasing solar luminosity for enhanced CO_2_ assimilation and/or energy production (72). The methane (CH_4_), metal sulfides, hydrogen (H_2_), and hydrogen sulfide (H_2_S) that are present in mining areas (47, 73) are also potential energy sources for *Acidiphilium*. Hydrogen (H_2_) might be formed in AMD areas through the acid dissolution of metals and minerals, and Ni/Fe hydrogenases might exploit this as an electron donor to support chemolithotrophic growth (74). Correspondingly, *Acidiphilium* acquired a nearly complete repertoire of methane and sulfur metabolic genes, as well as genes encoding Ni/Fe hydrogenase. Evidence of expression of the above-mentioned pathways has been shown in a *Rhodospirillales* relative (75). This evidence together with previously observed related phenotypes of *Acidiphilium* (see the introduction) suggests that these HGT-derived genes may also be functional in *Acidiphilium*. Numerous HGT-derived resistance genes for AMD adaption were present in *Acidiphilium*, similar to other AMD inhabitants (76, 77), reflecting diverse strategies of *Acidiphilium* to avert the deleterious effects of toxic metals and osmotic pressure in AMD environments. The acquired metabolic capacity of organic compounds and hydrocarbon in *Acidiphilium* suggested a mutualistic interaction of autotrophic acidophiles in AMD. For instance, metabolotoxic organic byproducts excreted by autotrophs (e.g., *Acidithiobacillus*) might be utilized by chemoorganotrophs (e.g., *Acidiphilium*), a process that in turn might stimulate the metabolic processes of the autotrophs (78–80). AMD microbial communities tend to form biofilms on mineral substrates for better metabolic cooperation and enhanced resistance against harsh environments (80–82). The microbes in biofilms are usually active, and the high community density, with increased proximity of microbes encapsulated in biofilm, might create more numerous opportunities for the efficient occurrence of HGT. In addition, MGEs, such as plasmids, might contribute to the development, stabilization, and expansion of biofilm (83–85). Consistent with this, our results showed that the predicted donors of HGT were also present in the cooccurrence network of *Acidiphilium.* Cooccurrence networks in microbial communities may provide hints for ecological interactions between species, on which HGT might have an influence (86, 87). Positive selection was also found to be an important driving force for adaptive evolution of *Acidiphilium.* Genes can be changed by positive selection for fixation of beneficial variants in a population/species over time if they increase survival fitness, which might help fine-tune gene expression in adaption to changing environmental conditions (27–30). Those genes under positive selection in *Acidiphilium* were prone to play key multifunctional roles, of which even small adaptive changes in their coding sequences might influence multiple pathways, bringing considerable benefits for survival of microbes during evolution in response to changing global conditions and shifting of niches. For example, the positively selected gene (PSG) *cysK* (Ap_2619, encoding cysteine synthase) might perform functions related to sulfide utilization, tellurite resistance, and growth inhibition (88–90); the PSG *pdxH* (Ap_2355), encoding pyridoxine/pyridoxamine 5′-phosphate oxidase, might act as a potent quencher of reactive oxygen intermediates and as an essential cofactor in amino acid metabolism (91, 92); and, finally, the PSG *trxA* (Ap_2636) encodes thioredoxin, a small redox protein that may play important roles in electron transfer, transcriptional regulation, immune response, and oxidative stress defense (93–96). However, further experiments are required to confirm their actual functions in *Acidiphilium*. The genus *Acidiphilium* is one of only four genera in the family *Acetobacteraceae* reported to colonize metal-rich AMD sites thus far, with the other genera of *Acetobacteraceae* found primarily in more moderate environments such as vinegar production environments and breweries (38, 97, 98). We suggest that the ancestor of *Acidiphilium* may have originated in mild or moderate conditions but then adapted to extreme environments, such as AMD niches, with the help of HGT and probable positive selection on the genes, similar to what has been found in acidophilic archaeal lineages such as *Thermoplasmatales* and *Sulfolobales*, which seemed to have evolved independently from moderately acidophilic ancestors (99). It is foreseeable that as more *Acidiphilium* strains are isolated and sequenced, the panorama of *Acidiphilium* evolution will gradually unfold before us.

### Conclusions.

Extremophiles that thrive in extremely acidic environments are research model organisms for microbial adaption and evolution. In this study, we provided evidence that *Acidiphilium* is characterized by a complex lifestyle granted by HGT. By way of gene acquisitions, *Acidiphilium* has greatly expanded its genetic diversity, resulting in functional divergence. *Acidiphilium* has acquired multiple abilities via HGT, such as photosynthesis, CO_2_ assimilation, metal resistance, and organic compound metabolism, which would facilitate beneficial interactions with cohabitant autotrophs. In addition, the predicted donors of HGT were present in the cooccurrence network of *Acidiphilium*. Positive selection on new mutations was also an important driving force in the evolution of *Acidiphilium*. We further proposed that microorganisms originating under mild conditions can adapt to extreme environments such as AMD sites after the acquisition of multiple adaptive functions. Taken together, this study has shed light on the ecological roles and evolutionary scenario of *Acidiphilium* and is a good example of research on the adaption and evolution of extremophiles.

## MATERIALS AND METHODS

### DNA extraction, genome sequencing, and assembly.

Strains AccI and AccII were isolated from an acid mine drainage (AMD) water sample obtained in the Mangzi mining area (formed by oxidizing dissolution of pyrite, characterized by a high ferric ion concentration), Yunnan Province, China (long 103.5, lat 23.3, altitude 1,847 m). Strain ZJSH63 was isolated from an AMD water sample obtained in the heap leaching area for copper ore in the Zijinshan Gold and Copper Mine, Fujian Province (lat 25.2, long 116.4, altitude 282.6 m), China. Genomic DNA of strains AccI, AccII, and ZJSH63 were extracted using the Qiagen genomic DNA extraction kit (Qiagen, Hilden, Germany) in accordance with the manufacturer's instructions. After the DNA sample passed quality testing, the large fragments were subjected to agarose recovery using a BluePippin automatic nucleic acid recovery instrument (SAGE Science). The DNA was damaged and repaired; after purification, the DNA fragments were end repaired and linked with adenine. After a purification and ligation reaction, Qubit was used to accurately quantify the constructed DNA library by following official protocol (http://cshprotocols.cshlp.org/content/2017/6/pdb.prot094730). The DNA library was subjected to the PacBio Sequel platform for sequencing at Guangdong Magigene Biotechnology Co. Ltd. (Guangzhou, China). After sequencing, SMRT Link v5.1.0 (https://www.pacb.com/support/software-downloads/) was utilized for correction and assembly.

### ANI and whole-genome alignments.

JSpecies v1.2.1 was used to calculate average nucleotide identity (ANI) based on the BLASTN algorithm with default parameters (100). BLASTN-based whole-genome comparison of *Acidiphilium* strains (completeness > 97%) was performed and represented with BRIG-0.95 (101). We utilized Circos (102) for construction and visualization of the multiple genome alignments of strains with completely sequenced genomes, including AccI, JF-5, and AIU301.

### Pangenome analyses and gene family evolution analyses of *Acidiphilium*.

A summary of features for the *Acidiphilium* genomes involved in this study are listed in Table 1. BUSCO (103) was used to estimate the completeness of each genome against its bacterial core gene set. Gene family clustering of 12 *Acidiphilium* genomes (completeness > 97%) together with UniProt search, GO Slim annotation, and GO enrichment analyses (default cutoff *P* value, 0.05) was performed via OrthoVenn2 (104) with default parameters. The BPGA pipeline (105) was used to perform model extrapolations of the *Acidiphilium* pangenome/core genome by applying default parameters. We applied COUNT (106) under the Wagner parsimony algorithm for ancestor genome size estimation and for detecting the gain, loss, expansion, and contraction events of gene families with the penalty ratio set to 1.

### Phylogenic analyses and divergence time estimation.

Phylogenetic trees based on 133 concatenated core genes and 16S rRNA gene sequences of *Acidiphilium* were constructed with the neighbor-joining (NJ) method using MEGA-X (107) with 1,000 bootstrap replicates. A chronogram for *Acidiphilium* species with branch lengths reflecting divergence times was inferred on the core gene tree of *Acidiphilium* using the RelTime method (108) implemented in MEGA-X with the JTT matrix-based model as described previously (109). The TimeTree reference data (110) that integrated data of asteroid impacts (Earth Impact Database, http://www.impact-structures.com/database-of-earth-impact-structures/), solar luminosity (111), and fluctuations in atmospheric O_2_ (112) and CO_2_ (113–116) amount were displayed synchronously with divergence times in the form of time panels. Phylogenetic trees based on protein sequences of functional genes were constructed using PhyML (117) with the maximum likelihood (ML) method and 1,000 bootstrap replicates, followed by visualization with iTOL (118). Sequences were aligned with Muscle (119) and trimmed with Gblocks (120) before tree construction.

### Genome annotation and horizontally transferred gene prediction.

We applied RAST (121), KEGG (122), and COG (123) databases (BLASTP cutoff, E value < 10^−5^) for genome annotation. We also extracted information of putative horizontally transferred genes from IMG Annotation results. Genome neighbor (context) visualizations were conducted with the EFI-GNT tool (124). Identification of putative horizontally transferred genes (HTGs) in the genomes of *Acidiphilium* was performed via the Integrated Microbial Genomes (IMG) system (125), which defined genes as being putative lateral transfers by the following principle: genes that have their best BLAST hits (best bit scores) or >90% of the best hits outside the taxonomic lineage of the genome (i.e., to genomes from another phylum, class, etc.) but with lower-scoring hits or no hits within the lineage.

### Prediction of mobile genetic elements.

We applied the ISfinder (126) to predict and classify insertion sequences (IS) and transposases within *Acidiphilium* genomes with BLASTP (cutoff E value, 1*e*^−5^). IslandViewer 4 (127) was used to detect putative genomic islands (GIs) distributed within *Acidiphilium* genomes. PHASTER (128) was applied to detect prophage and prophage remnant sequences within *Acidiphilium* genomes. We also applied CRISPRCasFinder (129) for detection of CRISPRs and Cas within *Acidiphilium* genomes.

### Construction of cooccurrence network.

To identify the associations between *Acidiphilium* and other microbes in AMD environments, 16S rRNA amplicon sequencing data sets of AMD samples (*n* = 205) were collected from the Sequence Read Archive (SRA) database (see Table S5 in the supplemental material). The QIIME (130) pipeline was applied to analyze these data sets. Sequences were clustered into operational taxonomic units (OTUs) at the 97% similarity level with the “closed reference OTU picking” strategy against the QIIME formatted Greengenes v.13.8 reference database (http://greengenes.lbl.gov). Rare OTUs, with fewer than five occurrences, were removed before network construction. The cooccurrence network was constructed using CoNet (131), which was implemented in Cytoscape v.3.6.1 based on the OTU occurrence frequency. Pairwise scores between OTUs were calculated using Spearman rank correlations applying a threshold rho of  >0.6 and a *P* value of <0.01. The cooccurrence network was visualized with Organic layout in Cytoscape v. 3.6.1 (132).

10.1128/mSystems.00867-20.5 TABLE S5 16S rRNA amplicon sequencing datasets of acid mine drainage (AMD) samples collected from the Sequence Read Archive (SRA) database for identification of the associations between *Acidiphilium* and other microbes in AMD environments. Download Table S5, XLSX file, 0.01 MB. Copyright © 2020 Li et al. 2020 Li et al. This content is distributed under the terms of the Creative Commons Attribution 4.0 International license.

### Genome-wide detection of positively selected genes.

We used the PosiGene pipeline (133) for genome-wide detection of positively selected genes in the above-mentioned strains of *Acidiphilium* spp., in which *Acidiphilium* sp. AccI was used as the anchor species, reference, and target species. Genes were considered under positive selection if the branch-wide test resulted in false discovery rates (FDR) of <0.05 and adjusted *P* values of <0.05.

### Data availability.

The genome sequences of *Acidiphilium* strains AccI, AccII, and ZJSH63 have been deposited in the JGI IMG-ER database under ER Genome IDs 2824045439, 2824049744, and 2828882166, respectively.

## References

[1] Merino N, Aronson HS, Bojanova DP, Feyhl-Buska J, Wong ML, Zhang S, Giovannelli D 2019 Living at the extremes: extremophiles and the limits of life in a planetary context. Front Microbiol 10:780. doi:10.3389/fmicb.2019.00780. 31037068 PMC6476344

[2] Rothschild LJ, Mancinelli RL 2001 Life in extreme environments. Nature 409:1092–1101. doi:10.1038/35059215. 11234023

[3] Hua ZS, Han YJ, Chen LX, Liu J, Hu M, Li SJ, Kuang JL, Chain PS, Huang LN, Shu WS 2015 Ecological roles of dominant and rare prokaryotes in acid mine drainage revealed by metagenomics and metatranscriptomics. ISME J 9:1280–1294. doi:10.1038/ismej.2014.212. 25361395 PMC4438317

[4] Kadnikov VV, Ivasenko DA, Beletsky AV, Mardanov AV, Danilova EV, Pimenov NV, Karnachuk OV, Ravin NV 2016 Effect of metal concentration on the microbial community in acid mine drainage of a polysulfide ore deposit. Microbiology 85:745–751. doi:10.1134/S0026261716060126. 28853774

[5] Kuang J, Huang L, He Z, Chen L, Hua Z, Jia P, Li S, Liu J, Li J, Zhou J, Shu W 2016 Predicting taxonomic and functional structure of microbial communities in acid mine drainage. ISME J 10:1527–1539. doi:10.1038/ismej.2015.201. 26943622 PMC5029178

[6] Kuang JL, Huang L, Chen L, Hua Z, Li S, Hu M, Li J, Shu W 2013 Contemporary environmental variation determines microbial diversity patterns in acid mine drainage. ISME J 7:1038–1050. doi:10.1038/ismej.2012.139. 23178673 PMC3635239

[7] Sievers M, Ludwig W, Teuber M 1994 Phylogenetic positioning of Acetobacter, Gluconobacter, Rhodopila and Acidiphilium species as a branch of acidophilic bacteria in the α-subclass of Proteobacteria based on 16S ribosomal DNA sequences. Syst Appl Microbiol 17:189–196. doi:10.1016/S0723-2020(11)80006-8.

[8] Cummings DE, Fendorf S, Singh N, Sani RK, Peyton BM, Magnuson TS 2007 Reduction of Cr(VI) under acidic conditions by the facultative Fe(III)-reducing bacterium *Acidiphilium cryptum*. Environ Sci Technol 41:146–152. doi:10.1021/es061333k. 17265940

[9] Küsel K, Dorsch T, Acker G, Stackebrandt E 1999 Microbial reduction of Fe(III) in acidic sediments: isolation of Acidiphilium cryptum JF-5 capable of coupling the reduction of Fe(III) to the oxidation of glucose. Appl Environ Microbiol 65:3633–3640. doi:10.1128/AEM.65.8.3633-3640.1999. 10427060 PMC91545

[10] Tomi T, Shibata Y, Ikeda Y, Taniguchi S, Haik C, Mataga N, Shimada K, Itoh S 2007 Energy and electron transfer in the photosynthetic reaction center complex of Acidiphilium rubrum containing Zn-bacteriochlorophyll a studied by femtosecond up-conversion spectroscopy. Biochim Biophys Acta 1767:22–30. doi:10.1016/j.bbabio.2006.10.008. 17169326

[11] Okamura K, Kawai A, Wakao N, Yamada T, Hiraishi A 2015 Acidiphilium iwatense sp. nov., isolated from an acid mine drainage treatment plant, and emendation of the genus Acidiphilium. Int J Syst Evol Microbiol 65:42–48. doi:10.1099/ijs.0.065052-0. 25273513

[12] Rohwerder T, Sand W 2003 The sulfane sulfur of persulfides is the actual substrate of the sulfur-oxidizing enzymes from Acidithiobacillus and Acidiphilium spp. Microbiology (Reading) 149:1699–1710. doi:10.1099/mic.0.26212-0. 12855721

[13] Xu A-l, Xia J-l, Song Z-w, Jiang P, Xia Y, Wan M-x, Zhang R-y, Yang Y, Liu K-k 2013 The effect of energy substrates on PHB accumulation of Acidiphilium cryptum DX1-1. Curr Microbiol 67:379–387. doi:10.1007/s00284-013-0373-y. 23657849

[14] Fischer JR, Quentmeier A, Gansel S, Sabados V, Friedrich CG 2002 Inducible aluminum resistance of Acidiphilium cryptum and aluminum tolerance of other acidophilic bacteria. Arch Microbiol 178:554–558. doi:10.1007/s00203-002-0482-7. 12420179

[15] Mahapatra NR, Banerjee PC 1996 Extreme tolerance to cadmium and high resistance to copper, nickel and zinc in different Acidiphilium strains. Lett Appl Microbiol 23:393–397. doi:10.1111/j.1472-765X.1996.tb01344.x.

[16] San Martin-Uriz P, Mirete S, Alcolea PJ, Gomez MJ, Amils R, Gonzalez-Pastor JE 2014 Nickel-resistance determinants in Acidiphilium sp. PM identified by genome-wide functional screening. PLoS One 9:e95041. doi:10.1371/journal.pone.0095041. 24740277 PMC3989265

[17] Borole AP, O’Neill H, Tsouris C, Cesar S 2008 A microbial fuel cell operating at low pH using the acidophile Acidiphilium cryptum. Biotechnol Lett 30:1367–1372. doi:10.1007/s10529-008-9700-y. 18368296

[18] Xu A-l, Xia J-l, Zhang S, Yang Y, Nie Z-y, Qiu G-z 2010 Bioleaching of chalcopyrite by UV-induced mutagenized Acidiphilium cryptum and Acidithiobacillus ferrooxidans. Trans Nonferrous Metals Soc China 20:315–321. doi:10.1016/S1003-6326(09)60140-0.

[19] González E, Rodríguez JM, Muñoz JÁ, Blázquez ML, Ballester A, González F 2018 The contribution of Acidiphilium cryptum to the dissolution of low-grade manganese ores. Hydrometallurgy 175:312–318. doi:10.1016/j.hydromet.2017.12.008.

[20] Priya A, Hait S 2017 Feasibility of bioleaching of selected metals from electronic; Waste by Acidiphilium acidophilum. Waste Biomass Valorization 9:871–877.

[21] Almeida WI, Vieira RP, Cardoso AM, Silveira CB, Costa RG, Gonzalez AM, Paranhos R, Medeiros JA, Freitas FA, Albano RM, Martins OB 2009 Archaeal and bacterial communities of heavy metal contaminated acidic waters from zinc mine residues in Sepetiba Bay. Extremophiles 13:263–271. doi:10.1007/s00792-008-0214-2. 19089530

[22] Hiraishi A, Matsuzawa Y, Kanbe T, Wakao N 2000 Acidisphaera rubrifaciens gen. nov., sp. nov., an aerobic bacteriochlorophyll-containing bacterium isolated from acidic environments. Int J Syst Evol Microbiol 50:1539–1546. doi:10.1099/00207713-50-4-1539. 10939661

[23] Jones RM, Hedrich S, Johnson DB 2013 Acidocella aromatica sp. nov.: an acidophilic heterotrophic alphaproteobacterium with unusual phenotypic traits. Extremophiles 17:841–850. doi:10.1007/s00792-013-0566-0. 23884710

[24] Liu Y, Yang H, Zhang X, Xiao Y, Guo X, Liu X 2016 Genomic analysis unravels reduced inorganic sulfur compound oxidation of heterotrophic acidophilic Acidicaldus sp. strain DX-1. BioMed Res Int 2016:8137012. doi:10.1155/2016/8137012. 27239474 PMC4864549

[25] Ren M, Feng X, Huang Y, Wang H, Hu Z, Clingenpeel S, Swan BK, Fonseca MM, Posada D, Stepanauskas R, Hollibaugh JT, Foster PG, Woyke T, Luo H 2019 Phylogenomics suggests oxygen availability as a driving force in Thaumarchaeota evolution. ISME J 13:2150–2161. doi:10.1038/s41396-019-0418-8. 31024152 PMC6776046

[26] Salcher MM, Schaefle D, Kaspar M, Neuenschwander SM, Ghai R 2019 Evolution in action: habitat transition from sediment to the pelagial leads to genome streamlining in Methylophilaceae. ISME J 13:2764–2777. doi:10.1038/s41396-019-0471-3. 31292537 PMC6794327

[27] Viana MVC, Sahm A, Goes Neto A, Figueiredo HCP, Wattam AR, Azevedo V 2018 Rapidly evolving changes and gene loss associated with host switching in Corynebacterium pseudotuberculosis. PLoS One 13:e0207304. doi:10.1371/journal.pone.0207304. 30419061 PMC6231662

[28] Petersen L, Bollback JP, Dimmic M, Hubisz M, Nielsen R 2007 Genes under positive selection in Escherichia coli. Genome Res 17:1336–1343. doi:10.1101/gr.6254707. 17675366 PMC1950902

[29] Props R, Monsieurs P, Vandamme P, Leys N, Denef VJ, Boon N 2019 Gene expansion and positive selection as bacterial adaptations to oligotrophic conditions. mSphere 4:e00011-19. doi:10.1128/mSphereDirect.00011-19. 30728279 PMC6365617

[30] Zhang Y, Jalan N, Zhou X, Goss E, Jones JB, Setubal JC, Deng X, Wang N 2015 Positive selection is the main driving force for evolution of citrus canker-causing Xanthomonas. ISME J 9:2128–2138. doi:10.1038/ismej.2015.15. 25689023 PMC4579464

[31] Bowers RM, Kyrpides NC, Stepanauskas R, Harmon-Smith M, Doud D, Reddy TBK, Schulz F, Jarett J, Rivers AR, Eloe-Fadrosh EA, Tringe SG, Ivanova NN, Copeland A, Clum A, Becraft ED, Malmstrom RR, Birren B, Podar M, Bork P, Weinstock GM, Garrity GM, Dodsworth JA, Yooseph S, Sutton G, Glöckner FO, Gilbert JA, Nelson WC, Hallam SJ, Jungbluth SP, Ettema TJG, Tighe S, Konstantinidis KT, Liu W-T, Baker BJ, Rattei T, Eisen JA, Hedlund B, McMahon KD, Fierer N, Knight R, Finn R, Cochrane G, Karsch-Mizrachi I, Tyson GW, Rinke C, Lapidus A, Meyer F, Yilmaz P, Parks DH, Eren AM, The Genome Standards Consortium, 2017 Minimum information about a single amplified genome (MISAG) and a metagenome-assembled genome (MIMAG) of bacteria and archaea. Nat Biotechnol 35:725–731. doi:10.1038/nbt.3893. 28787424 PMC6436528

[32] Jain C, Rodriguez-R LM, Phillippy AM, Konstantinidis KT, Aluru S 2018 High throughput ANI analysis of 90K prokaryotic genomes reveals clear species boundaries. Nat Commun 9:5114. doi:10.1038/s41467-018-07641-9. 30504855 PMC6269478

[33] Harrison AP 1981 Acidiphilium cryptum gen. nov., sp. nov., heterotrophic bacterium from acidic mineral environments. Int J Syst Bacteriol 31:327–332. doi:10.1099/00207713-31-3-327.

[34] Wakao N, Nagasawa N, Matsuura T, Matsukura H, Matsumoto T, Hiraishi A, Sakurai Y, Shiota H 1994 Acidiphilium multivorum sp. nov., an acidophilic chemoorganotrophic bacterium from pyritic acid mine drainage. J Gen Appl Microbiol 40:143–159. doi:10.2323/jgam.40.143.

[35] Wichlacz PL, Unz RF, Langworthy TA 1986 Acidiphilium angustum sp. nov., Acidiphilium facilis sp. nov., and Acidiphilium rubrum sp. nov.: acidophilic heterotrophic bacteria isolated from acidic coal mine drainage. Int J Syst Bacteriol 36:197–201. doi:10.1099/00207713-36-2-197.

[36] Medini D, Donati C, Tettelin H, Masignani V, Rappuoli R 2005 The microbial pan-genome. Curr Opin Genet Dev 15:589–594. doi:10.1016/j.gde.2005.09.006. 16185861

[37] Springael D, Top EM 2004 Horizontal gene transfer and microbial adaptation to xenobiotics: new types of mobile genetic elements and lessons from ecological studies. Trends Microbiol 12:53–58. doi:10.1016/j.tim.2003.12.010. 15040322

[38] De Roos J, Verce M, Aerts M, Vandamme P, De Vuyst L 2018 Temporal and spatial distribution of the acetic acid bacterium communities throughout the wooden casks used for the fermentation and maturation of lambic beer underlines their functional role. Appl Environ Microbiol 11:e02846-17. doi:10.1128/AEM.02846-17. PMC5861831 29352086

[39] Barrangou R, Fremaux C, Deveau H, Richards M, Boyaval P, Moineau S, Romero DA, Horvath P 2007 CRISPR provides acquired resistance against viruses in prokaryotes. Science 315:1709–1712. doi:10.1126/science.1138140. 17379808

[40] Varble A, Meaden S, Barrangou R, Westra ER, Marraffini LA 2019 Recombination between phages and CRISPR-cas loci facilitates horizontal gene transfer in staphylococci. Nat Microbiol 4:956–963. doi:10.1038/s41564-019-0400-2. 30886355 PMC6533911

[41] Konstantinidis KT, Tiedje JM 2004 Trends between gene content and genome size in prokaryotic species with larger genomes. Proc Natl Acad Sci U S A 101:3160–3165. doi:10.1073/pnas.0308653100. 14973198 PMC365760

[42] Govantes F, Albrecht JA, Gunsalus RP 2000 Oxygen regulation of the Escherichia coli cytochrome d oxidase (cydAB) operon: roles of multiple promoters and the Fnr‐1 and Fnr‐2 binding sites. Mol Microbiol 37:1456–1469. doi:10.1046/j.1365-2958.2000.02100.x. 10998176

[43] Sousa FL, Alves RJ, Ribeiro MA, Pereira-Leal JB, Teixeira M, Pereira MM 2012 The superfamily of heme-copper oxygen reductases: types and evolutionary considerations. Biochim Biophys Acta 1817:629–637. doi:10.1016/j.bbabio.2011.09.020. 22001780

[44] Chadwick GL, Hemp J, Fischer WW, Orphan VJ 2018 Convergent evolution of unusual complex I homologs with increased proton pumping capacity: energetic and ecological implications. ISME J 12:2668–2680. doi:10.1038/s41396-018-0210-1. 29991762 PMC6194058

[45] Palomo A, Pedersen AG, Fowler SJ, Dechesne A, Sicheritz-Pontã T, Smets BF 2018 Comparative genomics sheds light on niche differentiation and the evolutionary history of comammox Nitrospira. ISME J 12:1779–1793. doi:10.1038/s41396-018-0083-3. 29515170 PMC6018701

[46] Sharma P, Mattos MJTD, Hellingwerf KJ, Bekker M 2012 On the function of the various quinone species in Escherichia coli. FEBS J 279:3364–3373. doi:10.1111/j.1742-4658.2012.08608.x. 22521170

[47] Ozmen I, Aksoy E 2015 Respiratory emergencies and management of mining accidents. Turk Thorac J 16(Suppl 1):S18–S20. 29404110 10.5152/ttd.2015.005 PMC5783100

[48] Xie J, He Z, Liu X, Liu X, Van Nostrand JD, Deng Y, Wu L, Zhou J, Qiu G 2011 GeoChip-based analysis of the functional gene diversity and metabolic potential of microbial communities in acid mine drainage. Appl Environ Microbiol 77:991–999. doi:10.1128/AEM.01798-10. 21097602 PMC3028740

[49] Magnuson TS, Swenson MW, Paszczynski AJ, Deobald LA, Kerk D, Cummings DE 2010 Proteogenomic and functional analysis of chromate reduction in Acidiphilium cryptum JF-5, an Fe(III)-respiring acidophile. Biometals 23:1129–1138. doi:10.1007/s10534-010-9360-y. 20593301

[50] Xue XM, Yan Y, Xu HJ, Wang N, Zhang X, Ye J 2014 ArsH from Synechocystis sp. PCC 6803 reduces chromate and ferric iron. FEMS Microbiol Lett 356:105–112. doi:10.1111/1574-6968.12481. 24861149

[51] Rehan M, Furnholm T, Finethy RH, Chu F, El-Fadly G, Tisa LS 2014 Copper tolerance in Frankia sp. strain EuI1c involves surface binding and copper transport. Appl Microbiol Biotechnol 98:8005–8015. doi:10.1007/s00253-014-5849-6. 24903815

[52] Renninger N, Knopp R, Nitsche H, Clark DS, Keasling JD 2004 Uranyl precipitation by Pseudomonas aeruginosa via controlled polyphosphate metabolism. Appl Environ Microbiol 70:7404–7412. doi:10.1128/AEM.70.12.7404-7412.2004. 15574942 PMC535141

[53] Khaleque HN, Gonzalez C, Shafique R, Kaksonen AH, Holmes DS, Watkin ELJ 2019 Uncovering the mechanisms of halotolerance in the extremely acidophilic members of the Acidihalobacter genus through comparative genome analysis. Front Microbiol 10:155. doi:10.3389/fmicb.2019.00155. 30853944 PMC6396713

[54] Roesser M, Müller V 2010 Osmoadaptation in bacteria and archaea: common principles and differences. Environ Microbiol 3:743–754. doi:10.1046/j.1462-2920.2001.00252.x. 11846768

[55] Moritz KD, Amendt B, Witt EMHJ, Galinski EA 2015 The hydroxyectoine gene cluster of the non-halophilic acidophile Acidiphilium cryptum. Extremophiles 19:87–99. doi:10.1007/s00792-014-0687-0. 25142158

[56] Welte C, Hafner S, Krätzer C, Quentmeier A, Friedrich CG, Dahl C 2009 Interaction between Sox proteins of two physiologically distinct bacteria and a new protein involved in thiosulfate oxidation. FEBS Lett 583:1281–1286. doi:10.1016/j.febslet.2009.03.020. 19303410

[57] Wu W, Pang X, Lin J, Liu X, Wang R, Lin J, Chen L 2017 Discovery of a new subgroup of sulfur dioxygenases and characterization of sulfur dioxygenases in the sulfur metabolic network of Acidithiobacillus caldus. PLoS One 12:e0183668. doi:10.1371/journal.pone.0183668. 28873420 PMC5584763

[58] Denkmann K, Grein F, Zigann R, Siemen A, Bergmann J, van Helmont S, Nicolai A, Pereira IAC, Dahl C 2012 Thiosulfate dehydrogenase: a widespread unusual acidophilic c-type cytochrome. Environ Microbiol 14:2673–2688. doi:10.1111/j.1462-2920.2012.02820.x. 22779704

[59] Murrell JC, Smith TJ 2010 Biochemistry and molecular biology of methane monooxygenase *In* Timmis KN (ed), Handbook of hydrocarbon and lipid microbiology. Springer, Berlin, Germany.

[60] Wang VC, Maji S, Chen PP, Lee HK, Yu SS, Chan SI 2017 Alkane oxidation: methane monooxygenases, related enzymes, and their biomimetics. Chem Rev 117:8574–8621. doi:10.1021/acs.chemrev.6b00624. 28206744

[61] AAAS. 2005 So much more to know. Science 309:78–102. doi:10.1126/science.309.5731.78b. 15994524

[62] Chouaia B, Gaiarsa S, Crotti E, Comandatore F, Esposti MD, Ricci I, Alma A, Favia G, Bandi C, Daffonchio D 2014 Acetic acid bacteria genomes reveal functional traits for adaptation to life in insect guts. Genome Biol Evol 6:912–920. doi:10.1093/gbe/evu062. 24682158 PMC4007555

[63] Li L, Illeghems K, Van Kerrebroeck S, Borremans W, Cleenwerck I, Smagghe G, De Vuyst L, Vandamme P 2016 Whole-genome sequence analysis of Bombella intestini LMG 28161T, a novel acetic acid bacterium isolated from the crop of a red-tailed bumble bee, Bombus lapidarius. PLoS One 11:e0165611. doi:10.1371/journal.pone.0165611. 27851750 PMC5112900

[64] Bentkowski P, Van Oosterhout C, Mock T 2015 A model of genome size evolution for prokaryotes in stable and fluctuating environments. Genome Biol Evol 7:2344–2351. doi:10.1093/gbe/evv148. 26242601 PMC4558865

[65] Guieysse B, Wuertz S 2012 Metabolically versatile large-genome prokaryotes. Curr Opin Biotechnol 23:467–473. doi:10.1016/j.copbio.2011.12.022. 22226959

[66] Bentkowski P, van Oosterhout C, Ashby B, Mock T 2017 The effect of extrinsic mortality on genome size evolution in prokaryotes. ISME J 11:1011–1018. doi:10.1038/ismej.2016.165. 27922601 PMC5364348

[67] Brown BP, Wernegreen JJ 2019 Genomic erosion and extensive horizontal gene transfer in gut-associated Acetobacteraceae. BMC Genomics 20:472. doi:10.1186/s12864-019-5844-5. 31182035 PMC6558740

[68] Vos M, Hesselman MC, Te Beek TA, van Passel MWJ, Eyre-Walker A 2015 Rates of lateral gene transfer in prokaryotes: high but why? Trends Microbiol 23:598–605. doi:10.1016/j.tim.2015.07.006. 26433693

[69] Lindsey ARI, Newton ILG 2019 Some like it HOT: horizontal operon transfer. Cell 176:1243–1245. doi:10.1016/j.cell.2019.02.007. 30849369

[70] Brinkmann H, Göker M, Koblížek M, Wagner-Döbler I, Petersen J 2018 Horizontal operon transfer, plasmids, and the evolution of photosynthesis in Rhodobacteraceae. ISME J 12:1994–2010. doi:10.1038/s41396-018-0150-9. 29795276 PMC6052148

[71] Yelton AP, Comolli LR, Justice NB, Castelle C, Denef VJ, Thomas BC, Banfield JF 2013 Comparative genomics in acid mine drainage biofilm communities reveals metabolic and structural differentiation of co-occurring archaea. BMC Genomics 14:485. doi:10.1186/1471-2164-14-485. 23865623 PMC3750248

[72] Kishimoto N, Fukaya F, Inagaki K, Sugio T, Tanaka H, Tano T 1995 Distribution of bacteriochlorophyll a among aerobic and acidophilic bacteria and light-enhanced CO2-incorporation in Acidiphilium rubrum. FEMS Microbiol Ecol 16:291–296. doi:10.1111/j.1574-6941.1995.tb00293.x.

[73] Ly T, Wright JR, Weit N, McLimans CJ, Ulrich N, Tokarev V, Valkanas MM, Trun N, Rummel S, Grant CJ, Lamendella R 2019 Microbial communities associated with passive acidic abandoned coal mine remediation. Front Microbiol 10:1955. doi:10.3389/fmicb.2019.01955. 31507566 PMC6716070

[74] Hedrich S, Johnson DB 2013 Aerobic and anaerobic oxidation of hydrogen by acidophilic bacteria. FEMS Microbiol Lett 349:40–45. doi:10.1111/1574-6968.12290. 24117601

[75] Orlova MV, Tarlachkov SV, Dubinina GA, Belousova EV, Tutukina MN, Grabovich MY 2016 Genomic insights into metabolic versatility of a lithotrophic sulfur-oxidizing diazotrophic alphaproteobacterium Azospirillum thiophilum. FEMS Microbiol Ecol 92:fiw199. doi:10.1093/femsec/fiw199. 27660606

[76] Li L, Liu Z, Meng D, Liu X, Li X, Zhang M, Tao J, Gu Y, Zhong S, Yin H 2019 Comparative genomic analysis reveals distribution, organization, and evolution of metal resistance genes in the genus Acidithiobacillus. Appl Environ Microbiol 85:e02153-18. doi:10.1128/AEM.02153-18. 30389769 PMC6328783

[77] Navarro CA, Von Bernath D, Jerez CA 2013 Heavy metal resistance strategies of acidophilic bacteria and their acquisition: importance for biomining and bioremediation. Biol Res 46:363–371. doi:10.4067/S0716-97602013000400008. 24510139

[78] Liu H, Yin H, Dai Y, Dai Z, Liu Y, Li Q, Jiang H, Liu X 2011 The co-culture of Acidithiobacillus ferrooxidans and Acidiphilium acidophilum enhances the growth, iron oxidation, and CO2 fixation. Arch Microbiol 193:857–866. doi:10.1007/s00203-011-0723-8. 21691775

[79] Muravyov M, Panyushkina A 2020 Distinct roles of acidophiles in complete oxidation of high-sulfur ferric leach product of zinc sulfide concentrate. Microorganisms 8:386. doi:10.3390/microorganisms8030386. PMC7143523 32164331

[80] Yang Y, Diao M, Liu K, Qian L, Nguyen AV, Qiu G 2013 Column bioleaching of low-grade copper ore by Acidithiobacillus ferrooxidans in pure and mixed cultures with a heterotrophic acidophile Acidiphilium sp. Hydrometallurgy 131–132:93–98. doi:10.1016/j.hydromet.2012.09.003.

[81] Denef VJ, Mueller RS, Banfield JF 2010 AMD biofilms: using model communities to study microbial evolution and ecological complexity in nature. ISME J 4:599–610. doi:10.1038/ismej.2009.158. 20164865

[82] Goltsman DSA, Comolli LR, Thomas BC, Banfield JF 2015 Community transcriptomics reveals unexpected high microbial diversity in acidophilic biofilm communities. ISME J 9:1014–1023. doi:10.1038/ismej.2014.200. 25361394 PMC4817702

[83] Ghigo JM 2001 Natural conjugative plasmids induce bacterial biofilm development. Nature 412:442–445. doi:10.1038/35086581. 11473319

[84] Molin S, Tolker-Nielsen T 2003 Gene transfer occurs with enhanced efficiency in biofilms and induces enhanced stabilisation of the biofilm structure. Curr Opin Biotechnol 14:255–261. doi:10.1016/S0958-1669(03)00036-3. 12849777

[85] Reisner A, Holler BM, Molin S, Zechner EL 2006 Synergistic effects in mixed Escherichia coli biofilms: conjugative plasmid transfer drives biofilm expansion. J Bacteriol 188:3582–3588. doi:10.1128/JB.188.10.3582-3588.2006. 16672612 PMC1482856

[86] Fang P, Peng F, Gao X, Xiao P, Yang J 2019 Decoupling the dynamics of bacterial taxonomy and antibiotic resistance function in a subtropical urban reservoir as revealed by high-frequency sampling. Front Microbiol 10:1448. doi:10.3389/fmicb.2019.01448. 31312186 PMC6614491

[87] Steele JA, Countway PD, Xia L, Vigil PD, Beman JM, Kim DY, Chow C-ET, Sachdeva R, Jones AC, Schwalbach MS, Rose JM, Hewson I, Patel A, Sun F, Caron DA, Fuhrman JA 2011 Marine bacterial, archaeal and protistan association networks reveal ecological linkages. ISME J 5:1414–1425. doi:10.1038/ismej.2011.24. 21430787 PMC3160682

[88] Ramirez A, Castañeda M, Xiqui ML, Sosa A, Baca BE 2010 Identification, cloning and characterization of cysK, the gene encoding O-acetylserine (thiol)-lyase from Azospirillum brasilense, which is involved in tellurite resistance. FEMS Microbiol Lett 261:272–279. doi:10.1111/j.1574-6968.2006.00369.x. 16907731

[89] Johnson PM, Beck CM, Morse RP, Garza-Sánchez F, Low DA, Hayes CS, Goulding CW 2016 Unraveling the essential role of CysK in CDI toxin activation. Proc Natl Acad Sci U S A 113:9792–9797. doi:10.1073/pnas.1607112113. 27531961 PMC5024621

[90] Kaundal S, Uttam M, Thakur KG 2016 Dual role of a biosynthetic enzyme, CysK, in contact dependent growth inhibition in bacteria. PLoS One 11:e0159844. doi:10.1371/journal.pone.0159844. 27458806 PMC4961446

[91] Ankisettypalli K, Cheng JJ, Baker EN, Bashiri G 2016 PdxH proteins of mycobacteria are typical members of the classical pyridoxine/pyridoxamine 5'-phosphate oxidase family. FEBS Lett 590:453–460. doi:10.1002/1873-3468.12080. 26823273

[92] Matxain JM, Padro D, Ristilä M, Strid A, Eriksson LA 2009 Evidence of high *OH radical quenching efficiency by vitamin B6. J Phys Chem B 113:9629–9632. doi:10.1021/jp903023c. 19558175

[93] Mosterz J, Hochgräfe F, Jürgen B, Schweder T, Hecker M 2010 The role of thioredoxin TrxA in Bacillus subtilis: a proteomics and transcriptomics approach. Proteomics 8:2676–2690. doi:10.1002/pmic.200701015. 18601268

[94] Lu J, Holmgren A 2014 The thioredoxin antioxidant system. Free Radic Biol Med 66:75–87. doi:10.1016/j.freeradbiomed.2013.07.036. 23899494

[95] Möller MC, Hederstedt L 2008 Extracytoplasmic processes impaired by inactivation of trxA (thioredoxin gene) in Bacillus subtilis. J Bacteriol 190:4660–4665. doi:10.1128/JB.00252-08. 18456801 PMC2446788

[96] Sun JS, Li YX, Li S 2012 Cynoglossus semilaevis thioredoxin: a reductase and an antioxidant with immunostimulatory property. Cell Stress Chaperones 17:445–455. doi:10.1007/s12192-012-0322-x. 22270611 PMC3368026

[97] De Roos J, De Vuyst L 2018 Acetic acid bacteria in fermented foods and beverages. Curr Opin Biotechnol 49:115–119. doi:10.1016/j.copbio.2017.08.007. 28863341

[98] Azuma Y, Hosoyama A, Matsutani M, Furuya N, Horikawa H, Harada T, Hirakawa H, Kuhara S, Matsushita K, Fujita N, Shirai M 2009 Whole-genome analyses reveal genetic instability of Acetobacter pasteurianus. Nucleic Acids Res 37:5768–5783. doi:10.1093/nar/gkp612. 19638423 PMC2761278

[99] Colman DR, Poudel S, Hamilton TL, Havig JR, Selensky MJ, Shock EL, Boyd ES 2018 Geobiological feedbacks and the evolution of thermoacidophiles. ISME J 12:225–236. doi:10.1038/ismej.2017.162. 29028004 PMC5739016

[100] Richter M, Rossello-Mora R 2009 Shifting the genomic gold standard for the prokaryotic species definition. Proc Natl Acad Sci U S A 106:19126–19131. doi:10.1073/pnas.0906412106. 19855009 PMC2776425

[101] Alikhan NF, Petty NK, Zakour NLB, Beatson SAA 2011 BLAST Ring Image Generator (BRIG): simple prokaryote genome comparisons. BMC Genomics 12:402. doi:10.1186/1471-2164-12-402. 21824423 PMC3163573

[102] Krzywinski M, Schein J, Birol I, Connors J, Gascoyne R, Horsman D, Jones SJ, Marra MA 2009 Circos: an information aesthetic for comparative genomics. Genome Res 19:1639–1645. doi:10.1101/gr.092759.109. 19541911 PMC2752132

[103] Simao FA, Waterhouse RM, Ioannidis P, Kriventseva EV, Zdobnov EM 2015 BUSCO: assessing genome assembly and annotation completeness with single-copy orthologs. Bioinformatics 31:3210–3212. doi:10.1093/bioinformatics/btv351. 26059717

[104] Yi W, Coleman-Derr D, Chen G, Yong QG 2015 OrthoVenn: a web server for genome wide comparison and annotation of orthologous clusters across multiple species. Nucleic Acids Res 43:W78–84. doi:10.1093/nar/gkv487. 25964301 PMC4489293

[105] Chaudhari NM, Gupta VK, Dutta C 2016 BPGA—an ultra-fast pan-genome analysis pipeline. Sci Rep 6:24373. doi:10.1038/srep24373. 27071527 PMC4829868

[106] Miklós CS 2010 Count: evolutionary analysis of phylogenetic profiles with parsimony and likelihood. Bioinformatics 26:1910–1912. doi:10.1093/bioinformatics/btq315. 20551134

[107] Kumar S, Stecher G, Li M, Knyaz C, Tamura K 2018 MEGA X: Molecular Evolutionary Genetics Analysis across computing platforms. Mol Biol Evol 35:1547–1549. doi:10.1093/molbev/msy096. 29722887 PMC5967553

[108] Tamura K, Tao Q, Kumar S 2018 Theoretical foundation of the RelTime method for estimating divergence times from variable evolutionary rates. Mol Biol Evol 35:1770–1782. doi:10.1093/molbev/msy044. 29893954 PMC5995221

[109] Beatriz M 2018 Estimating timetrees with MEGA and the TimeTree resource. Mol Biol Evol 35:2334–2342. doi:10.1093/molbev/msy133. 29931306

[110] Kumar S, Stecher G, Suleski M, Hedges SB 2017 TimeTree: a resource for timelines, timetrees, and divergence times. Mol Biol Evol 34:1812–1819. doi:10.1093/molbev/msx116. 28387841

[111] Gough DO 1981 Solar interior structure and luminosity variations. Sol Phys 74:21–34. doi:10.1007/BF00151270.

[112] Holland HD 2006 The oxygenation of the atmosphere and oceans. Philos Trans R Soc Lond B Biol Sci 361:903–915. doi:10.1098/rstb.2006.1838. 16754606 PMC1578726

[113] Beerling DJ, Royer DL 2011 Convergent cenozoic CO_2_ history. Nat Geosci 4:418–420. doi:10.1038/ngeo1186.

[114] Berner AR 1990 Atmospheric carbon dioxide levels over phanerozoic time. Science 249:1382–1386. doi:10.1126/science.249.4975.1382. 17812165

[115] Hessler AM, Lowe DR, Jones RL, Bird DK 2004 A lower limit for atmospheric carbon dioxide levels 3.2 billion years ago. Nature 428:736–738. doi:10.1038/nature02471. 15085128

[116] Petit JR, Jouzel J, Raynaud D, Barkov NI, Barnola J-M, Basile I, Bender M, Chappellaz J, Davis M, Delaygue G, Delmotte M, Kotlyakov VM, Legrand M, Lipenkov VY, Lorius C, PÉpin L, Ritz C, Saltzman E, Stievenard M 1999 Climate and atmospheric history of the past 420,000 years from the Vostok ice core, Antarctica. Nature 399:429–436. doi:10.1038/20859.

[117] Guindon S, Delsuc F, Dufayard JF, Gascuel O 2009 Estimating maximum likelihood phylogenies with PhyML. Methods Mol Biol 537:113–137. doi:10.1007/978-1-59745-251-9_6. 19378142

[118] Letunic I, Bork P 2016 Interactive tree of life (iTOL) v3: an online tool for the display and annotation of phylogenetic and other trees. Nucleic Acids Res 44:W242–W245. doi:10.1093/nar/gkw290. 27095192 PMC4987883

[119] Edgar RC 2004 MUSCLE: a multiple sequence alignment method with reduced time and space complexity. BMC Bioinformatics 5:113. doi:10.1186/1471-2105-5-113. 15318951 PMC517706

[120] Talavera G, Castresana J 2007 Improvement of phylogenies after removing divergent and ambiguously aligned blocks from protein sequence alignments. Syst Biol 56:564–577. doi:10.1080/10635150701472164. 17654362

[121] Aziz RK, Bartels D, Best AA, DeJongh M, Disz T, Edwards RA, Formsma K, Gerdes S, Glass EM, Kubal M, Meyer F, Olsen GJ, Olson R, Osterman AL, Overbeek RA, McNeil LK, Paarmann D, Paczian T, Parrello B, Pusch GD, Reich C, Stevens R, Vassieva O, Vonstein V, Wilke A, Zagnitko O 2008 The RAST server: rapid annotations using subsystems technology. BMC Genomics 9:75–75. doi:10.1186/1471-2164-9-75. 18261238 PMC2265698

[122] Kanehisa M, Sato Y, Furumichi M, Morishima K, Tanabe M 2019 New approach for understanding genome variations in KEGG. Nucleic Acids Res 47:D590–D595. doi:10.1093/nar/gky962. 30321428 PMC6324070

[123] Tatusov RL, Galperin MY, Natale DA, Koonin EV 2000 The COG database: a tool for genome-scale analysis of protein functions and evolution. Nucleic Acids Res 28:33–36. doi:10.1093/nar/28.1.33. 10592175 PMC102395

[124] Gerlt AJ 2017 Genomic enzymology: Web tools for leveraging protein family sequence-function space and genome context to discover novel functions. Biochemistry 56:4293–4308. doi:10.1021/acs.biochem.7b00614. 28826221 PMC5569362

[125] Markowitz VM, Chen IM, Palaniappan K, Chu K, Szeto E, Grechkin Y, Ratner A, Anderson I, Lykidis A, Mavromatis K 2009 The integrated microbial genomes system: an expanding comparative analysis resource. Nucleic Acids Res 38:D382–D390. 19864254 10.1093/nar/gkp887 PMC2808961

[126] Siguier P, Perochon J, Lestrade L, Mahillon J, Chandler M 2006 ISfinder: the reference centre for bacterial insertion sequences. Nucleic Acids Res 34:D32–D36. doi:10.1093/nar/gkj014. 16381877 PMC1347377

[127] Bertelli C, Laird MR, Williams KP, Lau BY, Hoad G, Winsor GL, Brinkman FS, Simon Fraser University Research Computing Group 2017 IslandViewer 4: expanded prediction of genomic islands for larger-scale datasets. Nucleic Acids Res 45:W30–W35. doi:10.1093/nar/gkx343. 28472413 PMC5570257

[128] Arndt D, Grant JR, Marcu A, Sajed T, Pon A, Liang Y, Wishart DS 2016 PHASTER: a better, faster version of the PHAST phage search tool. Nucleic Acids Res 44:W16–W21. doi:10.1093/nar/gkw387. 27141966 PMC4987931

[129] Couvin D, Bernheim A, Toffano-Nioche C, Touchon M, Michalik J, Néron B, Rocha EPC, Vergnaud G, Gautheret D, Pourcel C 2018 CRISPRCasFinder, an update of CRISRFinder, includes a portable version, enhanced performance and integrates search for Cas proteins. Nucleic Acids Res 46:W246–W251. doi:10.1093/nar/gky425. 29790974 PMC6030898

[130] Caporaso JG, Kuczynski J, Stombaugh J, Bittinger K, Bushman FD, Costello EK, Fierer N, Peña AG, Goodrich JK, Gordon JI, Huttley GA, Kelley ST, Knights D, Koenig JE, Ley RE, Lozupone CA, McDonald D, Muegge BD, Pirrung M, Reeder J, Sevinsky JR, Turnbaugh PJ, Walters WA, Widmann J, Yatsunenko T, Zaneveld J, Knight R 2010 QIIME allows analysis of high-throughput community sequencing data. Nat Methods 7:335–336. doi:10.1038/nmeth.f.303. 20383131 PMC3156573

[131] Faust K, Raes J 2016 CoNet app: inference of biological association networks using Cytoscape. F1000Res 5:1519. doi:10.12688/f1000research.9050.2. 27853510 PMC5089131

[132] Cline MS, Smoot M, Cerami E, Kuchinsky A, Landys N, Workman C, Christmas R, Avila-Campilo I, Creech M, Gross B, Hanspers K, Isserlin R, Kelley R, Killcoyne S, Lotia S, Maere S, Morris J, Ono K, Pavlovic V, Pico AR, Vailaya A, Wang P-L, Adler A, Conklin BR, Hood L, Kuiper M, Sander C, Schmulevich I, Schwikowski B, Warner GJ, Ideker T, Bader GD 2007 Integration of biological networks and gene expression data using Cytoscape. Nat Protoc 2:2366–2382. doi:10.1038/nprot.2007.324. 17947979 PMC3685583

[133] Sahm A, Bens M, Platzer M, Szafranski K 2017 PosiGene: automated and easy-to-use pipeline for genome-wide detection of positively selected genes. Nucleic Acids Res 45:e100. doi:10.1093/nar/gkx179. 28334822 PMC5499814

