# Supplementary material for: Insights into the Metabolism and Evolution of the Genus Acidiphilium, a Typical Acidophile in Acid Mine Drainage
Source: mSystems. 2020 Nov 17;5(6):e00867-20. doi: 10.1128/mSystems.00867-20 (PMC7677001; doi:10.1128/mSystems.00867-20)
Supplement: TEXT S1 [file mSystems.00867-20-s0001.docx]

**COG categories description**

J: Translation, ribosomal structure and biogenesis;

K: Transcription;

L: Replication, recombination and repair;

D: Cell cycle control, cell division, chromosome partitioning;

V: Defense mechanisms;

T: Signal transduction mechanisms;

M: Cell wall/membrane/envelope biogenesis;

N: Cell motility;

U: Intracellular trafficking, secretion, and vesicular transport;

O: Posttranslational modification, protein turnover, chaperones;

C: Energy production and conversion;

G: Carbohydrate transport and metabolism;

E: Amino acid transport and metabolism;

F: Nucleotide transport and metabolism;

H: Coenzyme transport and metabolism;

I: Lipid transport and metabolism;

P: Inorganic ion transport and metabolism;

X: Mobilome: prophages, transposons;

Q: Secondary metabolites biosynthesis, transport and catabolism;

R: General function prediction only;

S: Function unknown.
