# Supplementary material for: Insights into the Metabolism and Evolution of the Genus Acidiphilium, a Typical Acidophile in Acid Mine Drainage
Source: mSystems. 2020 Nov 17;5(6):e00867-20. doi: 10.1128/mSystems.00867-20 (PMC7677001; doi:10.1128/mSystems.00867-20)
Supplement: TABLE S4 [file mSystems.00867-20-st004.docx]

**Table S4** Genes in *Acidiphilium* identified as being under positive selection using Posigene pipeline.

|  |  |  |  |  |  |
| --- | --- | --- | --- | --- | --- |
| Gene locus | FDR | P-Value | COG  class | Gene  name | Product |
| Ap_279 | 8.88E-03 | 2.11E-04 | I | *aidB* | Acyl-CoA dehydrogenase related to the alkylation response protein |
| Ap_2355 | 8.88E-03 | 5.56E-04 | H | *pdxH* | Pyridoxine/pyridoxamine 5'-phosphate oxidase |
| Ap_2636 | 8.88E-03 | 6.15E-04 | O | *trxA* | Thiol-disulfide isomerase or thioredoxin |
| Ap_346 | 8.88E-03 | 9.19E-04 | P | *clcA* | H+/Cl- antiporter |
| Ap_2034 | 8.88E-03 | 9.65E-04 | X | COG2963 | Transposase and inactivated derivatives |
| Ap_1473 | 1.36E-02 | 1.77E-03 | C | *ydfG* | NADP-dependent 3-hydroxy acid dehydrogenase |
| Ap_1855 | 1.39E-02 | 2.13E-03 | E | *asnB* | Asparagine synthetase B (glutamine-hydrolyzing) |
| Ap_1029 | 1.39E-02 | 2.61E-03 | - | - | - |
| Ap_37 | 1.39E-02 | 2.71E-03 | K | *vacB* | Exoribonuclease R |
| Ap_1038 | 1.57E-02 | 3.42E-03 | K | COG0816 | RNase H-fold protein, predicted Holliday junction resolvase  involved in anti-termination at Rho-dependent terminators |
| Ap_2619 | 3.44E-02 | 8.23E-03 | E | *cysK* | Cysteine synthase |
| Ap_696 | 4.62E-02 | 1.21E-02 | S | COG1289 | Uncharacterized membrane protein |
| Ap_1766 | 4.64E-02 | 1.32E-02 | - | - | - |
| Ap_374 | 4.64E-02 | 1.41E-02 | P | *nrfD* | Formate-dependent nitrite reductase, membrane component |
| Ap_835 | 4.71E-02 | 1.54E-02 | J | *rimI* | Ribosomal protein S18 acetylase and related acetyltransferases |
|  |  |  |  |  |  |
